# Supplementary material for: Feasibility of a screening and prevention procedure for risks associated with dysphagia in older patients in geriatric units: the DYSPHAGING pilot study protocol
Source: BMJ Open. 2024 Apr 19;14(4):e081333. doi: 10.1136/bmjopen-2023-081333 (PMC11033636; doi:10.1136/bmjopen-2023-081333)
Supplement: Supplementary data [file bmjopen-2023-081333supp001.pdf]

Patient code : /__/ /__/ /__//__/ /__/ /__/ /__//__/
First letter : Last name then first name centre N° patient identification N°

Online supplementary document 1

ALLIED HEALTH PROFESSIONAL SECTION

Page 1 : Characteristics of the respondant

You are:

☐ Nurse

☐ Nursing Assistant

☐ Doctor

☐ Else : ……………………………………………………………………

Page 2 : Satisfaction questionnaire

<u>If you take the presentation of the study as a whole</u>

1- *Strongly disagree*
2- *Somewhat disagree*
3- *No opinion*
4- *Somewhat agree*
5- *Strongly agree*

	1	*2*	*3*	*4*	*5*
**Do you think the explanations are appropriate?**					
**Was the time allocated sufficient?**					
**Is the summary sheet clear?**					
**Do you think the illustrations are clear?**					

How would you rate the presentation session?
(useless = 0 ; very useful = 10) : ……………..

<u>Did you find the procedure (DYSPHAGING form) simple and feasible to carry out in your current practice?</u>

	1	*2*	*3*	*4*	*5*
**EAT-10 questionnaire ?**					
**Airway protection manœuvres ?**					
**Hygienic and dietary measures?**					
**Procedures for adapting textures?**					

How would you rate the DYSPHAGING form?
(useless = 0 ; very useful = 10) : …………

Patient code : /__/ /__/ /__//__/ /__/ /__/ /__//__/
First letter : Last name then first name centre N° patient identification N°

Have you encountered any difficulties?

1 *Not at all*
2 *Some*
3 *A lot*

	1	2	3
**when presentating to the patient the information leaflet?**			
**For informing the patient's entourage?**			
**For collecting oral consent?**			
**For carrying out the EAT-10 questionnaire?**			
**For carrying out protection manoeuvres?**			

Have you encountered any difficulties (questions concerning paramedical research)

1 *Not at all*
2 *Some*
3 *A lot*

	1	2	3
**when presentating to the patient the information leaflet?**			
**For informing the patient's entourage?**			
**For collecting oral consent?**			
**For carrying out the EAT-10 questionnaire?**			
**For carrying out protection manoeuvres?**			

Would you have liked more information? No ☐ Yes ☐
If so, which ones? ………
………
………
………

Which suggestions would you make to make the protocol more relevant to your practice?
……………………………………………………………………………………………………
………
……………………………………………………………………………………………………
