# Supplementary material for: Feasibility of a screening and prevention procedure for risks associated with dysphagia in older patients in geriatric units: the DYSPHAGING pilot study protocol
Source: BMJ Open. 2024 Apr 19;14(4):e081333. doi: 10.1136/bmjopen-2023-081333 (PMC11033636; doi:10.1136/bmjopen-2023-081333)
Supplement: Supplementary data [file bmjopen-2023-081333supp003.pdf]

Supplementary Table 2: World Health Organization Trial Registration Data Set.

**Data category**	**Information**[32]
Primary registry and trial identifying number	ClinicalTrials.gov<br>NCT05734586
Date of registration in primary registry	8 February, 2023
Secondary identifying numbers	69HCL22_0474
Source(s) of monetary or material support	Hospices Civils de Lyon, France
Primary sponsor	Hospices Civils de Lyon, France
Secondary sponsor(s)	N/A
Contact for public queries	Marion MERDINIAN, MD<br>Tel: 00 33 4 78 86 56 83<br>E-mail: marion.merdinian@chu-lyon.fr
Contact for scientific queries	Claire FALANDRY, MD, PhD<br>Tel: 00 33 4 78 86 66 34<br>E-mail: claire.falandry@chu-lyon.fr
Public title	Screening for Sarcopenic Dysphagia and the Implementation of Measures to Prevent Its Complications in Geriatric Patients [DYSPHAGING-PILOT]
Scientific title	Feasibility Study of Screening for Sarcopenic Dysphagia and the Implementation of Measures to Prevent Its Complications in Geriatric or Institutionalized Patients Aged ≥ 70 Years.
Countries of recruitment	France

**Data category**	**Information**[32]
Health condition(s) or problem(s) studied	Swallowing Disorder, Sarcopenic Dysphagia
Intervention(s)	Other: EAT-10 (Eating assessment Tool) screening questionnaire<br>After inclusion, issuance of the EAT-10 screening questionnaire for swallowing disorders by the healthcare team<br>Procedure: Protective measures for the upper airways<br>In the event of an EAT ≥2 score, immediate implementation or within three days by the healthcare team of protective measures for the upper airways in 3 sectors:<br>1: Postural maneuvers; 2: Hygienodietetic rules; 3: Food textures
Key inclusion and exclusion criteria	Inclusion Criteria:<br>• Patient aged ≥ 70 years,<br>• Patient affiliated to a social security system,<br>• Patient hospitalized in the health sector or in a medico-social institute,<br>• Patient informed of the study (information leaflet provided) and having orally signified their consent to inclusion in the study.<br>Exclusion Criteria:<br>• Patient unable to feed orally,<br>• Patient with an active pathology responsible for acute swallowing disorders (< 3 months) (neurodegenerative pathology with predominant motor impairment such as Charcot's disease, stroke, ENT disease).<br>• Patient unable to answer the questionnaire.
Study type	Interventional<br>Allocation: N/A<br>Intervention model: parallel assignment<br>Masking: None (Open Label)<br>Primary purpose: Other<br>Phase II
Date of first enrolment	June 1st,2023
Target sample size	102
Recruitment status	Recruiting
Primary outcome(s)	Proportion of complete achievement of steps 1 and 2 [Time Frame: Three days]<br>The judgment criterion is validated if<br>1. Stage 1 is performed and the EAT-10 < 2 or if<br>2. Stage 1 is performed with an EAT-10 ≥ 2 and stage 2 is performed within 3 days after stage 1.

**Data category**	**Information**[32]
Key secondary outcomes	• Percentage of eligible patients refusing to participate in the study [Time Frame: 18 months]<br>○ Number of eligible patients who refused to participate in the study<br>• Age, gender, comorbidities (CIRS-G), autonomy (ADL, IADL), co-medications [Time Frame: 19 months]<br>○ Patient characteristics will be collected at each site at the end of the study by a clinical research assistant based on their medical records.<br>• Rate of partial completion of the protocol [Time Frame: 19 months]<br>○ Proportion of non-performance of step 1 and/or step 2 within the time limit. Proportion of steps 2 carried out incompletely), description of the reasons<br>• Diagnosis of undernutrition and/or neurocognitive disorders and/or patent lung infection and/or COPD described in the patient's medical file, nutritional risk situation assessed by the Mini Nutritional Assessment® (MNA) [Time Frame: 19 months]<br>○ Patient characteristics will be collected at each site at the end of the study by a clinical research assistant based on their medical records.<br>• Composition and disciplines of the care team [Time Frame: 19 months]<br>○ At the end of the study, all data on the each allied health professionals will be collected on the dysphaging sheet<br>• Satisfaction of the care team (Likert scale). [Time Frame: 19 months]<br>○ At the end of the study, each allied health professionals who has been involved in the care of at least one patient will fill out a satisfaction questionnaire.
