# Supplementary material for: Feasibility of a screening and prevention procedure for risks associated with dysphagia in older patients in geriatric units: the DYSPHAGING pilot study protocol
Source: BMJ Open. 2024 Apr 19;14(4):e081333. doi: 10.1136/bmjopen-2023-081333 (PMC11033636; doi:10.1136/bmjopen-2023-081333)
Supplement: Author's manuscript [file bmjopen-2023-081333.draft_revisions.pdf]

BMJ Open

BMJ Open is committed to open peer review. As part of this commitment we make the peer review history of every article we publish publicly available.

When an article is published we post the peer reviewers' comments and the authors' responses online. We also post the versions of the paper that were used during peer review. These are the versions that the peer review comments apply to.

The versions of the paper that follow are the versions that were submitted during the peer review process. They are not the versions of record or the final published versions. They should not be cited or distributed as the published version of this manuscript.

BMJ Open is an open access journal and the full, final, typeset and author-corrected version of record of the manuscript is available on our site with no access controls, subscription charges or pay-per-view fees (http://bmjopen.bmj.com).

If you have any questions on BMJ Open's open peer review process please email info.bmjopen@bmj.com

BMJ Open

Feasibility of a screening and prevention procedure for risks associated with dysphagia in older patients in geriatric units: the DYSPHAGING pilot study protocol

Journal:	*BMJ Open*
Manuscript ID	bmjopen-2023-081333
Article Type:	Protocol
Date Submitted by the Author:	25-Oct-2023
Complete List of Authors:	Durlach, Olivier; Hospices Civils de Lyon, Institut du Vieillissement<br>Tripoz-dit-Masson, Stéphanie; Hospices Civils de Lyon, Centre de Recherche Clinique Vieillissement Cerveau Fragilité, Hôpital des Charpennes<br>Massé-Deragon, Nicolas; Hospices Civils de Lyon, Institut du Vieillissement<br>SUBTIL, Fabien; Universite Claude Bernard Lyon 1, CNRS, UMR5558, Laboratoire de Biométrie et Biologie Evolutive, Lyon; Hospices Civils de Lyon, Service de Biostatistique<br>Niasse-Sy, Zeinabou; Université Lyon 1 Faculte de Medecine et de Maieutique Lyon-Sud Charles Merieux, Geriatrics; Hospices Civils de Lyon<br>HERLEDAN, Chloé; Hospices Civils de Lyon, Unité de Pharmacie clinique oncologique; Université Claude Bernard Lyon 1, EA 3738 CICLY<br>Guittard, Laure; Hospices Civils de Lyon, Pôle de Santé Publique, Service Recherche et Epidémiologie cliniques; Université Claude Bernard Lyon 1, Research on Healthcare Performance (RESHAPE), Inserm U1290<br>Goldet, Karine; Hospices Civils de Lyon, Centre de Recherche Clinique Vieillissement Cerveau Fragilité, Hôpital des Charpennes<br>Merazga, Salima; Hospices Civils de Lyon, Direction à la Recherche en Santé<br>Chabert, Margaux; Hospices Civils de Lyon, Direction à la Recherche en Santé<br>Suel, Anne; Hospices Civils de Lyon, Direction à la Recherche en Santé<br>Merdinian, Marion; Hospices Civils de Lyon, Service de Gériatrie, Groupement Hospitalier Sud<br>Falandry, Claire; Hospices Civils de Lyon, Service de Gériatrie, Centre Hospitalier de la Croix-Rousse; University of Lyon, CarMeN Laboratory, Inserm U1060, INRA U1397, Université Claude Bernard Lyon 1, INSA Lyon, Charles Mérieux Medical School
Keywords:	GERIATRIC MEDICINE, NUTRITION & DIETETICS, Nursing Care, OTOLARYNGOLOGY, PUBLIC HEALTH

SCHOLARONE™
Manuscripts

Title

Feasibility of a screening and prevention procedure for risks associated with dysphagia in older patients in geriatric units: the DYSPHAGING pilot study protocol

Olivier Durlach[1], Stéphanie Tripoz-dit-Masson[2], Nicolas Massé-Deragon[1], Fabien Subtil[3,4], Zeinabou Niasse-Sy[5,6], Chloé Herlédan[7,8], Laure Guittard[9,10], Karine Goldet[2], Salima Merazga[11], Margaux Chabert[11], Anne Suel[11], Marion Merdinian[6], Claire Falandry[12,13]

1: Hospices Civils de Lyon, Lyon, FR

2: Hospices Civils de Lyon, Centre de Recherche Clinique Vieillissement Cerveau Fragilité, Hôpital des Charpennes, Villeurbanne, FR

3: Université Claude Bernard Lyon 1, CNRS, UMR5558, Laboratoire de Biométrie et Biologie Evolutive, Villeurbanne, FR

4: Hospices Civils de Lyon, Service de Biostatistique, Lyon, FR

5: Université Lyon 1 Faculté de Médecine et de Maïeutique Lyon-Sud Charles Merieux, Oullins, FR

6: Hospices Civils de Lyon, Groupement Hospitalier Sud, Service de Gériatrie, Pierre-Bénite, FR

7: Hospices Civils de Lyon, Unité de Pharmacie clinique oncologique, Pierre-Bénite, FR

8: Université Claude Bernard Lyon 1, EA 3738 CICLY, Oullins, FR

9: Hospices Civils de Lyon, Pôle de Santé Publique, Service Recherche et Epidémiologie cliniques, Lyon, FR

For peer review only

10: Université Claude Bernard Lyon 1, Research on Healthcare Performance (RESHAPE), Inserm U1290, Lyon, FR

11: Hospices Civils de Lyon, Direction à la Recherche en Santé, Lyon, FR

12: Hospices Civils de Lyon, Centre Hospitalier de la Croix-Rousse, Service de Gériatrie, Lyon, FR

13: Université de Lyon, CarMeN Laboratory, Inserm U1060, INRA U1397, Université Claude Bernard Lyon 1, INSA Lyon, Faculté de Médecine et de Maïeutique Lyon-Sud Charles Merieux, Oullins, FR

Correspondence: claire.falandry@chu-lyon.fr

Keywords: Geriatrics; dysphagia; sarcopenia; sarcopenic dysphagia; screening; pilot study

Word count: 3,707

For peer review only

Abstract

Background: Dysphagia, and particularly sarcopenic dysphagia is frequent in frail older patients. Sarcopenic dysphagia is a swallowing disorder caused by sarcopenia, corresponding to a loss of muscle mass and strength. It frequently leads to inhalation and to the decrease of food intake, leading the patient to enter a vicious circle of chronic malnutrition and frailty. The awareness of the major health impacts of sarcopenic dysphagia is recent, explaining a low rate of screening in the population at risk. In this context, methods of prevention, evaluation, and intervention of sarcopenic dysphagia adapted to the most at-risk population are necessary.

Methods: The DYSPHAGING pilot study is a prospective, multicenter, non-comparative study aiming to estimate the feasibility of an intervention on allied health professionals using the DYSPHAGING educational sheet designed to implement a 2-step procedure "screen – prevent" to prevent swallowing disorders related to sarcopenic dysphagia. After obtaining oral consent, patients are screened using EAT-10 score. In case of a score ≥2, procedures including positional maneuvers during mealtimes, food and texture adaptation should be implemented. The primary endpoint of the study is the feasibility of this 2-steps procedure (screening – prevention measures) in the first 3 days after patient's consent.

The study will include 102 patients, with an expected 10% of non-analyzable patients, recruited in acute geriatric wards, rehabilitation centers, and long term care units, with the hypothesis to reach a feasibility rate of 50% and reject a rate lower than 35%.

Ethics and dissemination: The study protocol was approved according French legislation (CPP Ile de France VII) on February 15, 2023. The results of the primary and secondary objectives will be published in peer-reviewed journals.

Trial registration number: NCT05734586.

Keywords: Geriatrics; dysphagia; sarcopenia; sarcopenic dysphagia; screening; pilot study

Strengths and limitations of this study

- The DYSPHAGING study is a pilot study focusing on geriatric patients in different care sectors.
- This study is based on a screening questionnaire recognized and used for the evaluation and follow-up of patients who benefit from rehabilitation and preventive measures of swallowing disorders complications.
- The DYSPHAGING study is a prospective pilot study that aims to estimate the feasibility of this intervention.
- Particular attention will be paid to the satisfaction of the nursing teams involved in the implementation of the questionnaire.

Introduction

Background and rationale

Sarcopenic dysphagia(1) is a swallowing disorder (or oropharyngeal dysphagia, OD) resulting from the expression of sarcopenia, characterized by the loss of muscle mass and strength due to age and chronic diseases, in the oropharyngeal tract.. This condition gives rise to critical complications related to inhalation risks(2,3) and exacerbates chronic undernutrition(4), creating a detrimental cycle. Although recent awareness of the high prevalence of sarcopenic dysphagia and its severe consequences among older individuals with disabilities and hospitalized patients has grown, the screening rate within the affected population remains low. In response, there is a pressing need for tailored prevention, assessment, and intervention methods specifically designed for this vulnerable demographic.

To address this issue, the European Society for Swallowing Disorders and the European Union Geriatric Medicine Society(1), have jointly developed a Dysphagia Working Group and published a white paper considering OD as a geriatric syndrome . This position paper advocated

for increased awareness of swallowing disorders, utilization of screening scores, preventive measures, standardized diagnostics, and implementation of targeted interventions.

In adherence to these recommendations, we have collaboratively developed a pedagogical tool, entitled DYSPHAGING, within our multidisciplinary unit, following a comprehensive four-step approach: 1) Screening, 2) Protection, 3) Diagnosis confirmation, and 4) Rehabilitation. The DYSPHAGING form was designed to allow, in routine care, a rapid screening and protection procedure. Using standardized questionnaires and a simple, and schematic iconography, it is expected to be handled in routine by nurses, care assistants and even caregivers. As a first step, the DYSPHAGING pilot study was designed to evaluate the feasibility of this screening and protection in diverse geriatric wards (acute care, rehabilitation, and long-term care units).

Methods and analysis

Objectives

Primary objective

The primary objective of DYSPHAGING pilot is to assess the feasibility of implementing steps 1 and 2 of the DYSPHAGING form in hospital care units in the three days after the patient's inclusion in the protocol.

Secondary objectives

Secondary objectives include measurement of the percentage of patients eligible and refusing to participate in the study, characterization of the target population (demographic and geriatric characteristics), quantification of non-implementation of protocol steps and reasons, description of factors associated with the risk of sarcopenic dysphagia, description of care team

review only

characteristics, satisfaction of the involved allied health professionals with the program and difficulties encountered for its implementation.

Trial design

DYSPHAGING pilot study is a prospective, non-comparative multicentre study conducted in three different geriatric wards at the university hospital of Lyon (Hospices Civils de Lyon).

Study sites and participants

The study population will include older patient identified either during their admission (in acure care and rehabilitation units) or during systematic assessments in long-term care units.

Inclusion criteria are: age ≥70 years, patient affiliated to an health system, informed of the study (information notice given) and having verbally indicated his/her non-objection to inclusion in the study.

Exclusion criteria are: patient either unable to be fed orally, or with an active pathology responsible for acute swallowing disorders (< 3 months): neurodegenerative pathology with predominant motor impairment such as Charcot disease, stroke, ear nose and throat pathology, patient under court protection, with progressive somatic or psychiatric pathologies that would impair his/her ability to perform study assessments, or for whom data collection is not possible.

Premature study exit criteria are: refusal to continue the study, transfer to another department within 3 days of screening, death. Data already collected will be kept and analyzed.

Intervention

The DYSPHAGING form was designed as a simple, clear, schematic, and pedagogic recto-verso datasheet to be easily handle in routine care (figure 1). The recto face contains the rapid Eating Assessment Tool (EAT-10)(5,6), proposed by the Dysphagia Working Group as one of

the most promising screening tools, as it is a self-reported questionnaire, shown to be internally consistent, reproducible, and valid[1]A cut off score of ≥2 was chosen as Rofes et al. demonstrated that it offers 89% sensitivity and 82% specificity for OD(7). The verso face contains three protection fields: postural maneuvers, dietary and health rules and adaptation of food textures according to the standardized tool developed by the International Dysphagia Diet Standardization Initiative (IDDSI)(8). The design of the form was developed multidisciplinary with dieticians and a particular attention was paid to the clarity and the understandability of the different schemas.

Following the transmission of an information notice and obtaining an oral consent from patients, the intervention involves the integration of patients into a structured screening and care process for sarcopenic dysphagia. The study aims to evaluate the ability of local caregivers, including nursing assistants and nurses in geriatric wards, to adhere to current screening recommendations and implement preventive measures in a routine and standardized manner. Additionally, patient characteristics will be collected at each site through a clinical research assistant (CRA) based on comprehensive medical records. Characteristics of the healthcare team and their satisfaction with the DYSPHAGING form will be assessed during this designated visit.

The intervention process consists of two steps: Step 1: recto face of the DYSPHAGING form, consisting of the EAT-10 swallowing disorder screening questionnaire; in case of a score <2, the patient is considered fit for routine care without any additional protection measures; in case of a score ≥2, the step 2 should be engaged within 3 days by the healthcare team to implement upper airway protection measures within the three protection fields (verso face of the DYSPHAGING form).

Patient characteristics will be collected at each site by a CRA based on comprehensive medical records. Characteristics of the healthcare team and their satisfaction with the DYSPHAGING educational sheet will be assessed during this designated visit.

Outcomes and measurements

The primary outcome of the study is the proportion of patients who fully complete steps 1 and 2 of the protocol. The endpoint is validated if either:

- Step 1 is completed and an EAT-10 score < 2, or
- Step 1 is completed with an EAT-10 score ≥2 and step 2 is completed within 3 days following step 1.

Secondary outcomes of the study include:

- The percentage of eligible patients who refuse to participate in the study,
- Patient characteristics, such as age, gender, comorbidities, functionality, and co-medications. Comorbidities will be assessed with the Cumulative Illness Rating Scale-Geriatric (CIRS-G); functionality according to the Activity Daily Life (ADL)(9) and Instrumental ADL (IADL)(10) scores; comedications will be described according to the galenic form and drug class prescribed.
- Description of the factors associated with the risk of sarcopenic dysphagia (malnutrition, defined as either a weight loss ≥5 % in the last 6 months or a body mass index (BMI) < 22kg/m²(11), patient at risk of malnutrition according to the mini-nutritional assessment (MNA) short form, neuro-cognitive disorders, active pulmonary infection, chronic obstructive pulmonary disease (COPD), nutritional risk situations).
- The rate of partial completion of the protocol.

For peer review only

- The composition and disciplines of the healthcare team, the level of satisfaction and the difficulties encountered by the involved allied health professionals. A structured questionnaire was specifically designed to evaluate both dimensions (Online supplementary document 1). Satisfaction will be explored Using Likert Scale questionnaires, counting 30 points concerning the initial presentation of the study to the healthcare team, 30 points concerning the feasibility to implement the protection interventions, 30 points concerning difficulties encountered during the study, and two open questions concerning any missing pieces of information or suggestion to improve the study.

Trial conduct

The conduct of the study is represented in Figure 2 and Table 1:

1) Implementation: Training by the principal investigator of the nursing teams at the investigation sites in the materials used in stages 1 and 2 of the DYSPHAGING protocol (EAT10, checklist of measures to prevent swallowing disorders)
2) Inclusion and screening

a) Inclusion: Information to the patient, collection of non-objection and verification of inclusion and non-inclusion criteria, collection of patient characteristics and clinical data.

b) On the same day as inclusion, performance of step 1 "Screening": dispensing of the 10-item EAT-10 screening questionnaire

3) If EAT-10 score < 2: End of patient participation
4) Completion of step 2 if EAT-10 score ≥ 2: Implementation (within three days of screening) by the health care team of upper airway protection measures appropriate to each patient.

Completion of the following checklist:

- Postural maneuvers (sitting eating, chin down, +/- head turned towards the paralysed limb, +/- double swallow, +/- Mendelsohn maneuver, +/- forced swallow, +/- (super)supraglottic swallow),
- Hygienic and dietary rules (eliminate risky foods, adapt fluids, take time, drink between sips, avoid distraction),
- Food textures (liquid, very slightly thick, slightly thick, moderately smooth/mixed smooth, mixed/pureed, ground, swallowing specific soft, normal).

5) Collection of the satisfaction and difficulties encountered by the involved allied health professionals with the program (online supplemental table 1).

Strategies for achieving adequate participant enrolment will regularly be implemented using formal (newsletters, posters, meetings) and informal methods to reach target sample size/

Sample size calculation

The program will be considered feasible, at the patient level, if the proportion of patients for whom steps 1 and 2 are achievable is statistically higher than 35%, with an anticipated proportion of 50% (= alternative hypothesis). Under theses hypotheses, and assuming 10% of patients that might be non-evaluable, the inclusion of 102 patients will be necessary to achieve 90% power to show that the program is feasible (one-sided alpha risk of 5%).The included patients will be analyzed according to the intention-to-treat principle.

Data management and statistical analyses

A CRA ensures proper study execution, data collection, and reporting. Inconsistencies will be reported to the study investigators in order to decide whether the data should be corrected or considered as missing. Adverse health events will be reported to regulatory authorities according to the legislation in force, provided they are aligned with the study's judgment criteria

(inhalation/aspiration pneumonia, weight loss, death from any cause). Any changes in the data will be reported. A detailed statistical analysis plan will be drafted before the database is frozen. It will take into account any changes in the protocol or unexpected events during the course of the study that have an impact on the analyses presented above. Planned analyses may be completed in line with the study objectives. The analyses will be carried out by an independent statistician with the latest version of the SAS version 9.4 (SAS Institute, Cary, North Carolina) and R (R Core Team. R Foundation for Statistical Computing, Vienna, Austria. URL https://www.R-project.org/) softwares environment. No intermediate analysis is scheduled.

<u>Descriptive analyses</u>

A flow diagram will describe the data available for the patient population at baseline and during each follow-up visit. Eligibility criteria for treated patients will be verified, as well as follow-up and end of study visits. Reasons for premature end of study will be provided. Characteristics of the study population, numbers and proportions of missing values will be reported. Patient characteristics will be described using mean and SD or median and IQR for quantitative variables, and frequencies and distribution for categorical variables. A comparison of baseline characteristics between patients with complete follow-up and those with attrition will be performed. Analyses will be performed on the available data, without imputation for missing data.

Primary analysis

The proportion of patients for whom steps 1 and 2 of the DYSPHAGING form in performed in the 3 days of inclusion will be assessed along with its corresponding 95% confidence interval. Patients for whom information on the completion of steps 1 and 2 is not available will be considered as not having completed these steps.

For peer review only

Secondary analyses

Analyses of the questionnaire for allied health professionals

Analyses will be performed independently using descriptive analyses for quantitative data using mean and SD or median and IQR for Likert scales; overt questions will be reported according to a flat analysis. The analysis of factors associated with sarcopenic dysphagia will be performed by logistic regression. Univariate analyses will be followed by multivariable analyses.

Confidentiality

Correspondence tables will be kept in a separate file that does not contain clinical data. The access to the nominative information is protected by a password, and confidentiality is guaranteed by the study.

Protocol amendments

Any important modification requiring a new ethics committee approval will be communicated in future publications. Any potential impact of protocol modifications on the results will be discussed as appropriate.

Trial status

Patient enrolment began on May 2023. Data are currently being collected.

Patient and public involvement

The information letter and consent form for the study were reviewed by a patient partner.

For peer review only

Discussion

Discussion of the intervention

Despite growing interest in screening for swallowing disorders, there is no standardized method on which consensus has been reached. Among the main limitations include the heterogenecity of its presentations, the large number of etiologies, sometimes the difficulty of accessing a speech therapist to confirm the diagnosis.

The aim of the DYSPHAGING approach is to bring together all the healthcare professionals involved in the patient's care, to ensure a multi-disciplinary approach and to use all the time spent with the patient to extract as much relevant information as possible. We believe that the screening and preventive measures proposed by this protocol are appropriate for the various geriatric sectors, despite the heterogeneity of the situations encountered in this population.

Discussion of the trial design

The main aim of this study is to assess the feasibility of screening and various preventive measures. The cutoff value of EAT10 of 2 was chosen to favor sensitivity over specificity, even if a recent meta-analysis argued for a better diagnostic accuracy with a cutoff value of 3, as the DYSPHAGING form was focused more on screening than diagnostic(12). It is therefore essential to gather information on the non-implementation of the first steps, to understand the obstacles to the adoption of these initiatives. Particular attention was paid to the satisfaction of care providers in giving feedback about their training and the work tool. Emphasis was placed on assessing their satisfaction and the ergonomics of the tools made available to them, using a dedicated questionnaire. As healthcare staff are at the center of diagnosis and care, it is essential to understand the barriers and obstacles they face, by assessing much feedback as possible.

The galenic formulation and drug class will also be analysed with care, as iatrogenicity is omnipresent in the geriatric population.

For peer review only

We hope to highlight the various difficulties encountered during this pilot study in order to draw the necessary conclusions for a larger-scale study.

Ethics and dissemination

The study sponsor is the Hospices Civils de Lyon, responsible for study insurance and pharmacovigilance. The study protocol (V1) was approved by the ethics committee on on February 15 2023 and covers all sites involved in this study.

The research will be carried out in accordance with the Helsinki Declaration and International Conference on Harmonisation-Good Clinical Practice Guidelines. The trial protocol fulfils the SPIRIT 2013 checklist (online supplementary table 1) and WHO trial registration data set (online supplementary table 2). The study complies with the principles of the data protection act in France and with the GDPR in force in Europe. Each investigator must collect an oral informed consent at the beginning of the procedure. This consent is retained in the patient's medical chart. The patient can stop participation in the study at any time with an oral instruction given to the investigator or CRA. Patients will be informed of additional amendments according to the law in force. The results of the primary and secondary objectives will be published in peer-reviewed journals. All authors of future publications will have to meet the criteria for authorship stated in the Uniform Requirements for Manuscripts Submitted to Biomedical Journals by the International Committee of Medical Journal Editors.

Declaration of interests

The authors declare that they have no conflicts of interest.

For peer review only

Access to data and Dissemination policy

The final data set of the DYSPHAGING pilot study will be available upon reasonable request after the publication of the primary objective. Data requests can be submitted to the corresponding author.

Ancillary and post-trial care

None.

Acknowledgements: The authors acknowledge the teams of Lyon Sud Hospital, Pierre Garraud Hospital who contribute to patient enrolment in this study. The authors would like to thank the Centre de Recherche Clinique (Clinical Research Center) Vieillissement Cerveau Fragilité and the Direction à la Recherche en Santé (Health Research Department) of the Hospices Civils de Lyon for their valuable help in trial design and conduct

Contributors: All authors participated to the trial design conception. DD and CF assumed fundraising and grant follow-up. OD led the drafting of the manuscript. All authors critically reviewed and approved the final version of the protocol.

Funding: This work was supported by the Institut Nutrition and the Fondation de l'Avenir (Grant N°MLHR2023-89).

Word count: 3,707

For peer review only

Figure 1: The DYSPHAGING Form (A: recto; B: verso)

A

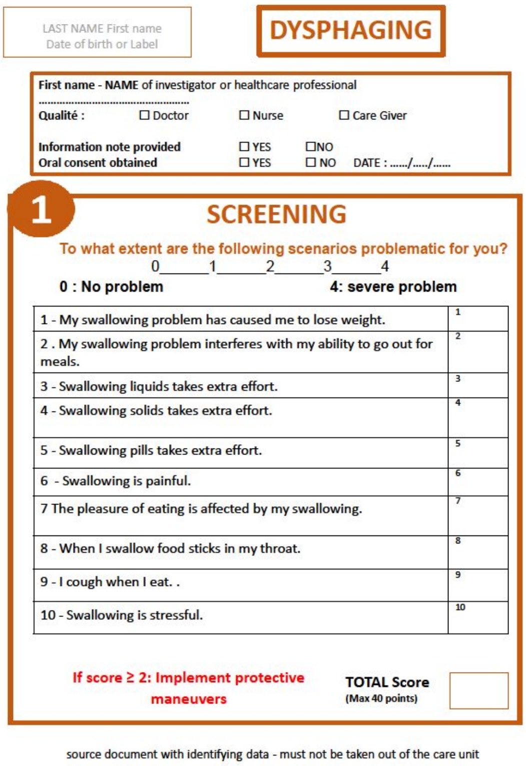

LAST NAME First name
Date of birth or Label

DYSPHAGING

First name - NAME of investigator or healthcare professional
....
Qualité : ☐ Doctor ☐ Nurse ☐ Care Giver

Information note provided ☐ YES ☐NO
Oral consent obtained ☐ YES ☐ NO DATE : ....../...../......

1 SCREENING

To what extent are the following scenarios problematic for you?

0____1____2____3____4

0 : No problem — 4: severe problem

1 - My swallowing problem has caused me to lose weight.	1
2 . My swallowing problem interferes with my ability to go out for meals.	2
3 - Swallowing liquids takes extra effort.	3
4 - Swallowing solids takes extra effort.	4
5 - Swallowing pills takes extra effort.	5
6 - Swallowing is painful.	6
7 The pleasure of eating is affected by my swallowing.	7
8 - When I swallow food sticks in my throat.	8
9 - I cough when I eat. .	9
10 - Swallowing is stressful.	10

If score ≥ 2: Implement protective maneuvers

TOTAL Score (Max 40 points)

source document with identifying data - must not be taken out of the care unit

B

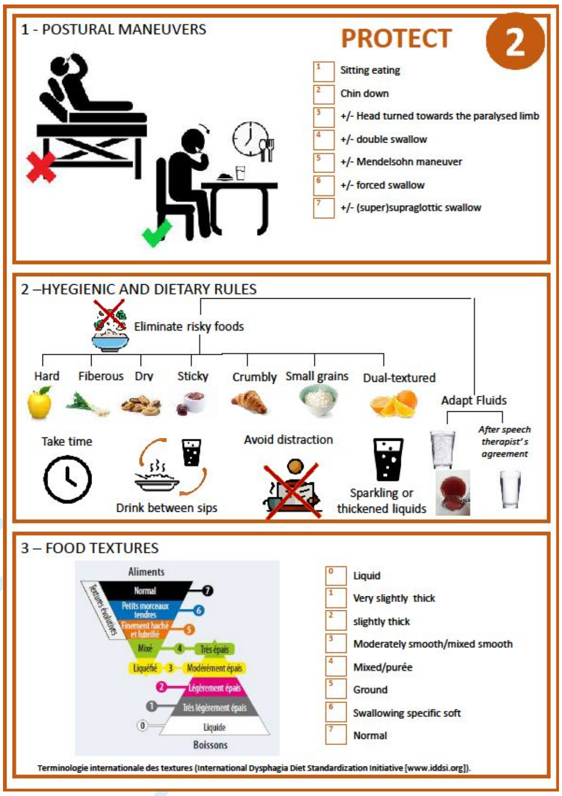

For peer review only

Figure 2: Design of the DYSPHAGING-pilot study

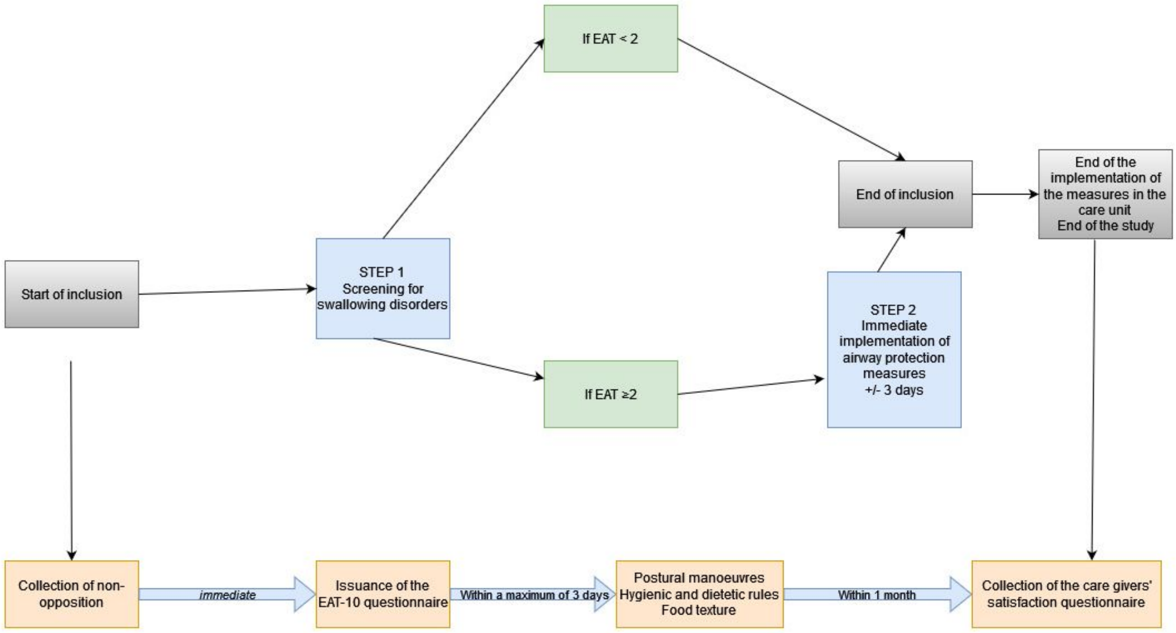

Table 1: DYSPHAGING-pilot study: flow diagram

Visits	V1	V2	End of the implementation of the measures
**Time of evaluation**	**Inclusion**	**End of inclusion**	**End of the study**
**PATIENT**			
Information notice	X		
Collection of non opposition	X		
Inclusion and exclusion criteria	X		
Population demographics[1]	X		
Nutritional risk factors[2]	X		
Functionnal independendence (ADL, IADL)	X		
Sarcopenic dysphagia risk factors[3]	X		
Sarcopenic dysphagia screening (EAT-10)	X		
Airway protection measures[4]		X	
**CARE GIVERS**			
Characteristics of the health care staff			X
Satisfaction questionnaire : Likert Scale			X

[1] Population demographics are age, gender, comorbidities (ICSR-G) and co-medications
[2] Nutritional risk factors are assessed by the Mini Nutritional Assessment® (MNA)
[3] Risk factors for sarcopenic dysphagia include undernutrition, neurocognitive impairment, overt lung infections and chronic obstructive pulmonary disease (COPD)
[4] Upper airway protection recommendations are validated by the following 3 methods: postural maneuvers, hygienic-dietary rules, textures within 3 days

For peer review only

References

1. Baijens LW, Clavé P, Cras P, Ekberg O, Forster A, Kolb G, et al. European Society for Swallowing Disorders – European Union Geriatric Medicine Society white paper: oropharyngeal dysphagia as a geriatric syndrome. Clin Interv Aging. oct 2016;Volume 11:1403‑28.

2. Yardimci B, Aksoy SM, Ozkaya I, Demir T, Tezcan G, Kaptanoglu AY. Anthropometric measurements may be informative for nursing home-acquired pneumonia. Pak J Med Sci [Internet]. 7 mai 2016 [cité 22 oct 2023];32(3). Disponible sur: http://pjms.com.pk/index.php/pjms/article/view/9635

3. Okazaki T, Ebihara S, Mori T, Izumi S, Ebihara T. Association between sarcopenia and pneumonia in older people. Geriatr Gerontol Int. janv 2020;20(1):7‑13.

4. Serra-Prat M, Palomera M, Gomez C, Sar-Shalom D, Saiz A, Montoya JG, et al. Oropharyngeal dysphagia as a risk factor for malnutrition and lower respiratory tract infection in independently living older persons: a population-based prospective study. Age Ageing. 1 mai 2012;41(3):376‑81.

5. Belafsky PC, Mouadeb DA, Rees CJ, Pryor JC, Postma GN, Allen J, et al. Validity and Reliability of the Eating Assessment Tool (EAT-10). Ann Otol Rhinol Laryngol. déc 2008;117(12):919‑24.

6. Lechien JR, Cavelier G, Thill MP, Huet K, Harmegnies B, Bousard L, et al. Validity and reliability of the French version of Eating Assessment Tool (EAT-10). Eur Arch Otorhinolaryngol. juin 2019;276(6):1727‑36.

7. Rofes L, Arreola V, Mukherjee R, Clavé P. Sensitivity and specificity of the Eating Assessment Tool and the Volume-Viscosity Swallow Test for clinical evaluation of oropharyngeal dysphagia. Neurogastroenterol Motil. sept 2014;26(9):1256‑65.

8. Cichero JAY, Lam P, Steele CM, Hanson B, Chen J, Dantas RO, et al. Development of International Terminology and Definitions for Texture-Modified Foods and Thickened Fluids Used in Dysphagia Management: The IDDSI Framework. Dysphagia. avr 2017;32(2):293‑314.

9. Katz S. Studies of Illness in the Aged: The Index of ADL: A Standardized Measure of Biological and Psychosocial Function. JAMA. 21 sept 1963;185(12):914.

10. Lawton MP, Brody EM. Assessment of older people: self-maintaining and instrumental activities of daily living. The Gerontologist. 1969;9(3):179‑86.

11. Diagnostic de la dénutrition chez la personne de 70 ans et plus. HAS 2021

12. Zhang P ping, Yuan Y, Lu D zhi, Li T ting, Zhang H, Wang H ying, et al. Diagnostic Accuracy of the Eating Assessment Tool-10 (EAT-10) in Screening Dysphagia: A Systematic Review and Meta-Analysis. Dysphagia. févr 2023;38(1):145‑58.

Patient code : /__/ /__/ /__//__/ /__/ /__/ /__//__/
First letter : Last name then first name centre N° patient identification N°

Online supplementary document 1

ALLIED HEALTH PROFESSIONAL SECTION

Page 1 : Characteristics of the professional

You are :

☐ Nurse

☐ Nursing Assistant

☐ Doctor

☐ Else : ………………………………………………………………………

Page 2 : Satisfaction questionnaire

<u>If you take the presentation of the study as a whole</u>

1- *Strongly disagree*
2- *Somewhat disagree*
3- *No opinion*
4- *Somewhat agree*
5- *Strongly agree*

	1	*2*	*3*	*4*	*5*
**Do you think the explanations are appropriate?**					
**Was the time allocated sufficient?**					
**Is the summary sheet clear?**					
**Do you think the illustrations are clear?**					

How would you rate the presentation session?
(useless = 0 ; very useful = 10) : ……………..

<u>Did you find the procedure (DYSPHAGING form) simple and feasible to carry out in your current practice?</u>

	1	*2*	*3*	*4*	*5*
**EAT-10 questionnaire ?**					
**Airway protection manœuvres ?**					
**Hygienic and dietary measures?**					
**Procedures for adapting textures?**					

How would you rate the DYSPHAGING form?
(useless = 0 ; very useful = 10) : …………

For peer review only

Have you encountered any difficulties

1 Not at all
2 Some
3 A lot

	1	2	3
**when presentating to the patient the information leaflet?**			
**For informing the patient's entourage?**			
**For collecting oral consent?**			
**For carrying out the EAT-10 questionnaire?**			
**For carrying out protection manoeuvres?**			

Have you encountered any difficulties (questions concerning paramedical research)

1 Not at all
2 Some
3 A lot

	1	2	3
**when presentating to the patient the information leaflet?**			
**For informing the patient's entourage?**			
**For collecting oral consent?**			
**For carrying out the EAT-10 questionnaire?**			
**For carrying out protection manoeuvres?**			

Would you have liked more information? No ☐ Yes ☐
If so, which ones,………
………
………
………

What suggestions would you make to make the protocol more relevant to your practice?………
……
………

For peer review only

Supplementary table 1: SPIR

SPIRIT 2013 Checklist: Recommended items to address in a clinical trial protocol and related documents*

Section/item	ItemNo	Description	Reported on page #
**Administrative information**			
Title	1	Descriptive title identifying the study design, population, interventions, and, if applicable, trial acronym	1
Trial registration	2a	Trial identifier and registry name. If not yet registered, name of intended registry	1
	2b	All items from the World Health Organization Trial Registration Data Set	Online supplementary table 2
Protocol version	3	Date and version identifier	N/A
Funding	4	Sources and types of financial, material, and other support	14 (Funding)
Roles and responsibilities	5a	Names, affiliations, and roles of protocol contributors	1 (Authors' list)<br>14 (Contributors)
	5b	Name and contact information for the trial sponsor	13 (ethics and dissemination)

For peer review only

	5c	Role of study sponsor and funders, if any, in study design; collection, management, analysis, and interpretation of data; writing of the report; and the decision to submit the report for publication, including whether they will have ultimate authority over any of these activities	13 (ethics and dissemination)
	5d	Composition, roles, and responsibilities of the coordinating centre, steering committee, endpoint adjudication committee, data management team, and other individuals or groups overseeing the trial, if applicable (see Item 21a for data monitoring committee)	9 (Data management and statistical analyses) 13 (Ethics and dissemination)
**Introduction**			
Background and rationale	6a	Description of research question and justification for undertaking the trial, including summary of relevant studies (published and unpublished) examining benefits and harms for each intervention	3
	6b	Explanation for choice of comparators	N/A
Objectives	7	Specific objectives or hypotheses	4
Trial design	8	Description of trial design including type of trial (eg, parallel group, crossover, factorial, single group), allocation ratio, and framework (eg, superiority, equivalence, noninferiority, exploratory)	4-5
**Methods: Participants, interventions, and outcomes**			
Study setting	9	Description of study settings (eg, community clinic, academic hospital) and list of countries where data will be collected. Reference to where list of study sites can be obtained	4-5

For peer review only

Eligibility criteria	10	Inclusion and exclusion criteria for participants. If applicable, eligibility criteria for study centres and individuals who will perform the interventions (eg, surgeons, psychotherapists)	5
Interventions	11a	Interventions for each group with sufficient detail to allow replication, including how and when they will be administered	5-6
	11b	Criteria for discontinuing or modifying allocated interventions for a given trial participant (eg, drug dose change in response to harms, participant request, or improving/worsening disease)	5
	11c	Strategies to improve adherence to intervention protocols, and any procedures for monitoring adherence (eg, drug tablet return, laboratory tests)	N/A
	11d	Relevant concomitant care and interventions that are permitted or prohibited during the trial	N/A
Outcomes	12	Primary, secondary, and other outcomes, including the specific measurement variable (eg, systolic blood pressure), analysis metric (eg, change from baseline, final value, time to event), method of aggregation (eg, median, proportion), and time point for each outcome. Explanation of the clinical relevance of chosen efficacy and harm outcomes is strongly recommended	6-7 (Outcomes and measurements)
Participant timeline	13	Time schedule of enrolment, interventions (including any run-ins and washouts), assessments, and visits for participants. A schematic diagram is highly recommended (see Figure)	15 (Figure 2)
Sample size	14	Estimated number of participants needed to achieve study objectives and how it was determined, including clinical and statistical assumptions supporting any sample size calculations	9 (Sample size calculation)

For peer review only - http://bmjopen.bmj.com/site/about/guidelines.xhtml

Recruitment	15	Strategies for achieving adequate participant enrolment to reach target sample size	N/A
**Methods: Assignment of interventions (for controlled trials)**			**N/A**
Allocation:			
Sequence generation	16a	Method of generating the allocation sequence (eg, computer-generated random numbers), and list of any factors for stratification. To reduce predictability of a random sequence, details of any planned restriction (eg, blocking) should be provided in a separate document that is unavailable to those who enrol participants or assign interventions	-
Allocation concealment mechanism	16b	Mechanism of implementing the allocation sequence (eg, central telephone; sequentially numbered, opaque, sealed envelopes), describing any steps to conceal the sequence until interventions are assigned	-
Implementation	16c	Who will generate the allocation sequence, who will enrol participants, and who will assign participants to interventions	-
Blinding (masking)	17a	Who will be blinded after assignment to interventions (eg, trial participants, care providers, outcome assessors, data analysts), and how	-
	17b	If blinded, circumstances under which unblinding is permissible, and procedure for revealing a participant's allocated intervention during the trial	-
**Methods: Data collection, management, and analysis**			

Data collection methods	18a	Plans for assessment and collection of outcome, baseline, and other trial data, including any related processes to promote data quality (eg, duplicate measurements, training of assessors) and a description of study instruments (eg, questionnaires, laboratory tests) along with their reliability and validity, if known. Reference to where data collection forms can be found, if not in the protocol	7,9, Table 1
	18b	Plans to promote participant retention and complete follow-up, including list of any outcome data to be collected for participants who discontinue or deviate from intervention protocols	N/A
Data management	19	Plans for data entry, coding, security, and storage, including any related processes to promote data quality (eg, double data entry; range checks for data values). Reference to where details of data management procedures can be found, if not in the protocol	9-10 (Data management and statistical analyses)
Statistical methods	20a	Statistical methods for analysing primary and secondary outcomes. Reference to where other details of the statistical analysis plan can be found, if not in the protocol	9-10 (Data management and statistical analyses)
	20b	Methods for any additional analyses (eg, subgroup and adjusted analyses)	N/A
	20c	Definition of analysis population relating to protocol non-adherence (eg, as randomised analysis), and any statistical methods to handle missing data (eg, multiple imputation)	10 (Descriptive analyses)
**Methods: Monitoring**			

Data monitoring	21a	Composition of data monitoring committee (DMC); summary of its role and reporting structure; statement of whether it is independent from the sponsor and competing interests; and reference to where further details about its charter can be found, if not in the protocol. Alternatively, an explanation of why a DMC is not needed	N/A
	21b	Description of any interim analyses and stopping guidelines, including who will have access to these interim results and make the final decision to terminate the trial	N/A
Harms	22	Plans for collecting, assessing, reporting, and managing solicited and spontaneously reported adverse events and other unintended effects of trial interventions or trial conduct	9 (Data management and statistical analyses)
Auditing	23	Frequency and procedures for auditing trial conduct, if any, and whether the process will be independent from investigators and the sponsor	N/A
**Ethics and dissemination**			
Research ethics approval	24	Plans for seeking research ethics committee/institutional review board (REC/IRB) approval	13 (Ethics and dissemination)
Protocol amendments	25	Plans for communicating important protocol modifications (eg, changes to eligibility criteria, outcomes, analyses) to relevant parties (eg, investigators, REC/IRBs, trial participants, trial registries, journals, regulators)	11 (Protocol amendments)
Consent or assent	26a	Who will obtain informed consent or assent from potential trial participants or authorised surrogates, and how (see Item 32)	13 (Ethics and dissemination)
	26b	Additional consent provisions for collection and use of participant data and biological specimens in ancillary studies, if applicable	N/A

For peer review only - http://bmjopen.bmj.com/site/about/guidelines.xhtml

Confidentiality	27	How personal information about potential and enrolled participants will be collected, shared, and maintained in order to protect confidentiality before, during, and after the trial	9, 11 (Confidentiality)
Declaration of interests	28	Financial and other competing interests for principal investigators for the overall trial and each study site	13 (Declaration of interests)
Access to data	29	Statement of who will have access to the final trial dataset, and disclosure of contractual agreements that limit such access for investigators	13-14 (Access to data and dissemination policy)
Ancillary and post-trial care	30	Provisions, if any, for ancillary and post-trial care, and for compensation to those who suffer harm from trial participation	N/A
Dissemination policy	31a	Plans for investigators and sponsor to communicate trial results to participants, healthcare professionals, the public, and other relevant groups (eg, via publication, reporting in results databases, or other data sharing arrangements), including any publication restrictions	13-14 (Access to data and dissemination policy)
	31b	Authorship eligibility guidelines and any intended use of professional writers	15 (Dissemination policy), N/A
	31c	Plans, if any, for granting public access to the full protocol, participant-level dataset, and statistical code	13-14 (Acces to data and dissemination policy)
**Appendices**			
Informed consent materials	32	Model consent form and other related documentation given to participants and authorised surrogates	Fig. 1 DYSPHAGING Form

For peer review only - http://bmjopen.bmj.com/site/about/guidelines.xhtml

Biological specimens	33	Plans for collection, laboratory evaluation, and storage of biological specimens for genetic or molecular analysis in the current trial and for future use in ancillary studies, if applicable	N/A

*It is strongly recommended that this checklist be read in conjunction with the SPIRIT 2013 Explanation & Elaboration for important clarification on the items. Amendments to the protocol should be tracked and dated. The SPIRIT checklist is copyrighted by the SPIRIT Group under the Creative Commons "Attribution-NonCommercial-NoDerivs 3.0 Unported" license.

For peer review only

Supplementary Table 2: World Health Organization Trial Registration Data Set.

**Data category**	**Information**[32]
Primary registry and trial identifying number	ClinicalTrials.gov<br>NCT05734586
Date of registration in primary registry	8 February, 2023
Secondary identifying numbers	69HCL22_0474
Source(s) of monetary or material support	Hospices Civils de Lyon, France
Primary sponsor	Hospices Civils de Lyon, France
Secondary sponsor(s)	N/A
Contact for public queries	Marion MERDINIAN, MD<br>Tel: 00 33 4 78 86 56 83<br>E-mail: marion.merdinian@chu-lyon.fr
Contact for scientific queries	Claire FALANDRY, MD, PhD<br>Numéro de téléphone: 00 33 4 78 86 66 34<br>E-mail: claire.falandry@chu-lyon.fr
Public title	Screening for Sarcopenic Dysphagia and the Implementation of Measures to Prevent Its Complications in Geriatric Patients [DYSPHAGING-PILOT]
Scientific title	Feasibility Study of Screening for Sarcopenic Dysphagia and the Implementation of Measures to Prevent Its Complications in Geriatric or Institutionalized Patients Aged ≥ 70 Years.
Countries of recruitment	France

For peer review only

**Data category**	**Information**[32]
Health condition(s) or problem(s) studied	Swallowing Disorder, Sarcopenic Dysphagia
Intervention(s)	Other: EAT-10 (Eating assessment Tool) screening questionnaire<br>After inclusion, issuance of the EAT-10 screening questionnaire for swallowing disorders by the healthcare team<br>Procedure: Protective measures for the upper airways<br>In the event of an EAT ≥2 score, immediate implementation or within three days by the healthcare team of protective measures for the upper airways in 3 sectors:<br>1: Postural maneuvers; 2: Hygienodietetic rules; 3: Food textures
Key inclusion and exclusion criteria	Inclusion Criteria:<br>• Patient aged ≥ 70 years,<br>• Patient affiliated to a social security system,<br>• Patient hospitalized in the health sector or in a medico-social institute,<br>• Patient informed of the study (information leaflet provided) and having orally signified their consent to inclusion in the study.<br>Exclusion Criteria:<br>• Patient unable to feed orally,<br>• Patient under legal protection, guardianship or curatorship,<br>• Patient with an active pathology responsible for acute swallowing disorders (< 3 months) (neurodegenerative pathology with predominant motor impairment such as Charcot's disease, stroke, ENT disease).<br>• Patient unable to answer the questionnaire.
Study type	Interventional<br>Allocation: N/A<br>Intervention model: parallel assignment<br>Masking: None (Open Label)<br>Primary purpose: Other<br>Phase II
Date of first enrolment	June 1st,2023
Target sample size	102
Recruitment status	Recruiting
Primary outcome(s)	Proportion of complete achievement of steps 1 and 2 [Time Frame: Three days]<br>The judgment criterion is validated if<br>1. Stage 1 is performed and the EAT-10 < 2 or if<br>2. Stage 1 is performed with an EAT-10 ≥ 2 and stage 2 is performed within 3 days after stage 1.

For peer review only

Data category	Information[32]
Key secondary outcomes	• Percentage of eligible patients refusing to participate in the study [Time Frame: 18 months]<br>○ Number of eligible patients who refused to participate in the study<br>• Age, gender, comorbidities (CIRS-G), autonomy (ADL, IADL), co-medications [Time Frame: 19 months]<br>○ Patient characteristics will be collected at each site at the end of the study by a clinical research assistant based on their medical records.<br>• Rate of partial completion of the protocol [Time Frame: 19 months]<br>○ Proportion of non-performance of step 1 and/or step 2 within the time limit. Proportion of steps 2 carried out incompletely), description of the reasons<br>• Diagnosis of undernutrition and/or neurocognitive disorders and/or patent lung infection and/or COPD described in the patient's medical file, nutritional risk situation assessed by the Mini Nutritional Assessment® (MNA) [Time Frame: 19 months]<br>○ Patient characteristics will be collected at each site at the end of the study by a clinical research assistant based on their medical records.<br>• Composition and disciplines of the care team [Time Frame: 19 months]<br>○ At the end of the study, all data on the each allied health professionals will be collected on the dysphaging sheet<br>• Caregiver satisfaction (Likert scale). [Time Frame: 19 months]<br>○ At the end of the study, each allied health professionals who has been involved in the care of at least one patient will fill out a satisfaction questionnaire.

E

For peer review only

BMJ Open

Feasibility of a screening and prevention procedure for risks associated with dysphagia in older patients in geriatric units: the DYSPHAGING pilot study protocol

Journal:	*BMJ Open*
Manuscript ID	bmjopen-2023-081333.R1
Article Type:	Protocol
Date Submitted by the Author:	02-Feb-2024

Complete List of Authors:	Durlach, Olivier; Hospices Civils de Lyon, Institut du Vieillissement<br>Tripoz-dit-Masson, Stéphanie; Hospices Civils de Lyon, Centre de Recherche Clinique Vieillissement Cerveau Fragilité, Hôpital des Charpennes<br>Massé-Deragon, Nicolas; Hospices Civils de Lyon, Institut du Vieillissement<br>SUBTIL, Fabien; Universite Claude Bernard Lyon 1, CNRS, UMR5558, Laboratoire de Biométrie et Biologie Evolutive, Lyon; Hospices Civils de Lyon, Service de Biostatistique<br>Niasse-Sy, Zeinabou; Université Lyon 1 Faculte de Medecine et de Maieutique Lyon-Sud Charles Merieux, Geriatrics; Hospices Civils de Lyon<br>HERLEDAN, Chloé; Hospices Civils de Lyon, Unité de Pharmacie clinique oncologique; Université Claude Bernard Lyon 1, EA 3738 CICLY<br>Guittard, Laure; Hospices Civils de Lyon, Pôle de Santé Publique, Service Recherche et Epidémiologie cliniques; Université Claude Bernard Lyon 1, Research on Healthcare Performance (RESHAPE), Inserm U1290<br>Goldet, Karine; Hospices Civils de Lyon, Centre de Recherche Clinique Vieillissement Cerveau Fragilité, Hôpital des Charpennes<br>Merazga, Salima; Hospices Civils de Lyon, Direction à la Recherche en Santé<br>Chabert, Margaux; Hospices Civils de Lyon, Direction à la Recherche en Santé<br>Suel, Anne; Hospices Civils de Lyon, Direction à la Recherche en Santé<br>Dayde, David; Hospices Civils de Lyon, Plateforme Transversale de Recherche de l'ICHCL<br>Merdinian, Marion; Hospices Civils de Lyon, Service de Gériatrie, Groupement Hospitalier Sud<br>Falandry, Claire; Hospices Civils de Lyon, Service de Gériatrie, Centre Hospitalier de la Croix-Rousse; University of Lyon, CarMeN Laboratory, Inserm U1060, INRA U1397, Université Claude Bernard Lyon 1, INSA Lyon, Charles Mérieux Medical School

**Primary Subject Heading**:	Geriatric medicine
Secondary Subject Heading:	Ear, nose and throat/otolaryngology, Nursing, Nutrition and metabolism
Keywords:	GERIATRIC MEDICINE, NUTRITION & DIETETICS, Nursing Care, OTOLARYNGOLOGY, PUBLIC HEALTH

SCHOLARONE™
Manuscripts

Title

Feasibility of a screening and prevention procedure for risks associated with dysphagia in older patients in geriatric units: the DYSPHAGING pilot study protocol

Olivier Durlach[1], Stéphanie Tripoz-dit-Masson[2], Nicolas Massé-Deragon[1], Fabien Subtil[3,4], Zeinabou Niasse-Sy[5,6], Chloé Herlédan[7,8], Laure Guittard[9,10], Karine Goldet[2], Salima Merazga[11], Margaux Chabert[11], Anne Suel[11], David Dayde[12], Marion Merdinian[6], Claire Falandry[13,14]

1: Hospices Civils de Lyon, Institut du Vieillissement, Lyon, FR

2: Hospices Civils de Lyon, Centre de Recherche Clinique Vieillissement Cerveau Fragilité, Hôpital des Charpennes, Villeurbanne, FR

3: Université Claude Bernard Lyon 1, CNRS, UMR5558, Laboratoire de Biométrie et Biologie Evolutive, Villeurbanne, FR

4: Hospices Civils de Lyon, Service de Biostatistique, Lyon, FR

5: Université Lyon 1 Faculté de Médecine et de Maïeutique Lyon-Sud Charles Merieux, Oullins, FR

6: Hospices Civils de Lyon, Groupement Hospitalier Sud, Service de Gériatrie, Pierre-Bénite, FR

7: Hospices Civils de Lyon, Unité de Pharmacie clinique oncologique, Pierre-Bénite, FR

8: Université Claude Bernard Lyon 1, EA 3738 CICLY, Oullins, FR

For peer review only

9: Hospices Civils de Lyon, Pôle de Santé Publique, Service Recherche et Epidémiologie cliniques, Lyon, FR

10: Université Claude Bernard Lyon 1, Research on Healthcare Performance (RESHAPE), Inserm U1290, Lyon, FR

11: Hospices Civils de Lyon, Direction à la Recherche en Santé, Lyon, FR

12: Hospices Civils de Lyon, Plateforme Transversale de Recherche en Cancérologie, Pierre-Bénite, FR

13: Hospices Civils de Lyon, Centre Hospitalier de la Croix-Rousse, Service de Gériatrie, Lyon, FR

14: Université de Lyon, CarMeN Laboratory, Inserm U1060, INRA U1397, Université Claude Bernard Lyon 1, INSA Lyon, Faculté de Médecine et de Maïeutique Lyon-Sud Charles Merieux, Oullins, FR

Correspondence: claire.falandry@chu-lyon.fr

Keywords: Geriatrics; dysphagia; sarcopenia; sarcopenic dysphagia; screening; pilot study

Word count: 3,619

For peer review only

Abstract

Background: Dysphagia, and particularly sarcopenic dysphagia is frequent in frail older patients. Sarcopenic dysphagia is a swallowing disorder caused by sarcopenia, corresponding to a loss of muscle mass and strength. It frequently leads to inhalation and to the decrease of food intake, leading the patient to enter a vicious circle of chronic malnutrition and frailty. The awareness of the major health impacts of sarcopenic dysphagia is recent, explaining a low rate of screening in the population at risk. In this context, methods of prevention, evaluation, and intervention of sarcopenic dysphagia adapted to the most at-risk population are necessary.

Methods: The DYSPHAGING pilot study is a prospective, multicenter, non-comparative study aiming to estimate the feasibility of an intervention on allied health professionals using the DYSPHAGING educational sheet designed to implement a 2-step procedure "screen – prevent" to prevent swallowing disorders related to sarcopenic dysphagia. After obtaining oral consent, patients are screened using EAT-10 score. In case of a score ≥2, procedures including positional maneuvers during mealtimes, food and texture adaptation should be implemented. The primary endpoint of the study is the feasibility of this 2-steps procedure (screening – prevention measures) in the first 3 days after patient's consent.

The study will include 102 patients, with an expected 10% of non-analyzable patients, recruited in acute geriatric wards, rehabilitation centers, and long-term care units, with the hypothesis to reach a feasibility rate of 50% and reject a rate lower than 35%.

Ethics and dissemination: The study protocol was approved according to French legislation (CPP Ile de France VII) on February 15, 2023. The results of the primary and secondary objectives will be published in peer-reviewed journals.

Trial registration number: NCT05734586.

Keywords: Geriatrics; dysphagia; sarcopenia; sarcopenic dysphagia; screening; pilot study

Strengths and limitations of this study

- The DYSPHAGING study is a pilot study focusing on geriatric patients in different care sectors.
- This study is based on a screening questionnaire recognized and used for the evaluation and follow-up of patients who benefit from rehabilitation and preventive measures of swallowing disorders complications.
- The DYSPHAGING study is a prospective pilot study that aims to estimate the feasibility of this intervention.
- Particular attention will be paid to the satisfaction of the nursing teams involved in the implementation of the questionnaire.

Introduction

Background and rationale

Sarcopenic dysphagia(1) is a swallowing disorder (or oropharyngeal dysphagia, OD) resulting from the expression of sarcopenia, characterized by the loss of muscle mass and strength due to age and chronic diseases, in the oropharyngeal tract.. This condition gives rise to critical complications related to inhalation risks (2,3) and exacerbates chronic undernutrition (4), creating a detrimental cycle. Although recent awareness of the high prevalence of sarcopenic dysphagia and its severe consequences among older individuals with disabilities and hospitalized patients has grown, the screening within the affected population remains low and challenging, leading to suboptimal care (5). In response, there is a pressing need for tailored prevention, assessment, and intervention methods specifically designed for this vulnerable demographic.

To address this issue, the European Society for Swallowing Disorders and the European Union Geriatric Medicine Society have jointly developed a Dysphagia Working Group and published

a white paper considering OD as a geriatric syndrome (1). This position paper advocated for increased awareness of swallowing disorders, utilization of screening scores, preventive measures, standardized diagnostics, and implementation of targeted interventions.

In adherence to these recommendations, we have collaboratively developed a pedagogical tool, entitled DYSPHAGING form, within our multidisciplinary unit, following a comprehensive four-step approach: 1) Screening, 2) Protection, 3) Diagnosis confirmation, and 4) Rehabilitation. The form was designed to allow, in routine care, a rapid screening and protection procedure. Using standardized questionnaires and a simple, and schematic iconography, it is expected to be handled in routine by nurses, care assistants and even caregivers. As a first step, the DYSPHAGING pilot study was designed to evaluate the feasibility of this screening and protection in diverse geriatric wards (acute care, rehabilitation, and long-term care units).

Methods and analysis

Objectives

Primary objective

The primary objective of DYSPHAGING pilot is to assess the feasibility of implementing steps 1 and 2 of the DYSPHAGING form in hospital care units in the three days after the patient's inclusion in the protocol.

Secondary objectives

Secondary objectives include measurement of the percentage of patients eligible and refusing to participate in the study, characterization of the target population (demographic and geriatric characteristics), quantification of non-implementation of protocol steps and reasons, description of factors associated with the risk of sarcopenic dysphagia, description of care team

For peer review only

characteristics, satisfaction of the involved allied health professionals with the program and difficulties encountered for its implementation.

Trial design

DYSPHAGING pilot study is a prospective, non-comparative multicentre study conducted in three different geriatric departments and two different hospitals at the university hospital of Lyon (Hospices Civils de Lyon).

Study sites and participants

The study population will include older patient identified either during their admission (in acure care and rehabilitation units) or during systematic assessments in long-term care units.

Inclusion criteria are: age ≥70 years, patient affiliated to an health system, informed of the study (information notice given) and having verbally indicated his/her non-objection to inclusion in the study.

Exclusion criteria are: patient either unable to be fed orally, or with an active pathology responsible for acute swallowing disorders (< 3 months): neurodegenerative pathology with predominant motor impairment such as Charcot disease, stroke, ear nose and throat pathology, patient under court protection, with progressive somatic or psychiatric pathologies that would impair his/her ability to perform study assessments, or for whom data collection is not possible.

Premature study exit criteria are: refusal to continue the study, transfer to another department within 3 days of screening, death. Data already collected will be kept and analyzed.

Intervention

The DYSPHAGING form was designed as a simple, clear, schematic, and pedagogic recto-verso form to be easily handle in routine care (figure 1). The recto face contains the rapid Eating

Assessment Tool (EAT-10) (6,7), proposed by the Dysphagia Working Group as one of the most promising screening tools, as it is a self-reported questionnaire, shown to be internally consistent, reproducible, and valid (1). A cut off score of ≥2 was chosen as Rofes et al. demonstrated that it offers 89% sensitivity and 82% specificity for OD (8). The verso face contains three protection fields: postural maneuvers, dietary and health rules and adaptation of food textures according to the standardized tool developed by the International Dysphagia Diet Standardization Initiative (IDDSI) (9). The design of the form was developed multidisciplinary with dieticians and a particular attention was paid to the clarity and the understandability of the different schemas.

Following the transmission of an information notice and obtaining an oral consent from patients (and their legal guardian for patients under guardianship) by either a physician or a paramedical professional under his/her responsibility, the intervention involves the integration of patients into a structured screening and care process for sarcopenic dysphagia. The study aims to evaluate the ability of local caregivers, including nursing assistants and nurses in geriatric wards, to adhere to current screening recommendations and implement preventive measures in a routine and standardized manner. Additionally, patient characteristics will be collected at each site through a clinical research assistant (CRA) based on comprehensive medical records. Characteristics of the healthcare team and their satisfaction with the DYSPHAGING form will be assessed during this designated visit.

The intervention process consists of two steps: Step 1: recto face of the DYSPHAGING form, consisting of the EAT-10 swallowing disorder screening questionnaire; in case of a score <2, the patient is considered fit for routine care without any additional protection measures; in case of a score ≥2, the step 2 should be engaged within 3 days by the healthcare team to implement upper airway protection measures within the three protection fields (verso face of the DYSPHAGING form).

Patient characteristics will be collected at each site by a CRA based on comprehensive medical records. Characteristics of the healthcare team and their satisfaction with the DYSPHAGING educational sheet will be assessed during this designated visit.

<u>Outcomes and measurements</u>

The primary outcome of the study is the proportion of patients who fully complete steps 1 and 2 of the protocol. The endpoint is validated if either:

- Step 1 is completed, and an EAT-10 score < 2, or
- Step 1 is completed with an EAT-10 score ≥2 and step 2 is completed within 3 days following step 1.

Secondary outcomes of the study include:

- The percentage of eligible patients who refuse to participate in the study,
- Patient characteristics, such as age, gender, comorbidities, functionality, and co-medications. Comorbidities will be assessed with the Cumulative Illness Rating Scale-Geriatric (CIRS-G); functionality according to the Activity Daily Life (ADL)(10) and Instrumental ADL (IADL)(11) scores; comedications will be described according to the galenic form and drug class prescribed.
- Description of the factors associated with the risk of sarcopenic dysphagia (malnutrition, defined as either a weight loss ≥5 % in the last 6 months or a body mass index (BMI) < 22kg/m²(12), patient at risk of malnutrition according to the mini-nutritional assessment (MNA) short form, neuro-cognitive disorders, active pulmonary infection, chronic obstructive pulmonary disease (COPD), nutritional risk situations).
- The rate of partial completion of the protocol.

For peer review only

- The composition and disciplines of the healthcare team, the level of satisfaction and the difficulties encountered by the involved allied health professionals. A structured questionnaire was specifically designed to evaluate both dimensions (Online supplementary document 1). Satisfaction will be explored Using Likert Scale questionnaires, counting 30 points concerning the initial presentation of the study to the healthcare team, 30 points concerning the feasibility to implement the protection interventions, 30 points concerning difficulties encountered during the study, and two open questions concerning any missing pieces of information or suggestion to improve the study.

<u>Trial conduct</u>

The conduct of the study is represented in Figure 2 and Table 1:

1) Implementation: Training by the principal investigator of the nursing teams at the investigation sites in the materials used in stages 1 and 2 of the DYSPHAGING protocol (EAT10, checklist of measures to prevent swallowing disorders)
2) Inclusion and screening

a) Inclusion: Information to the patient is provided by either the physician or a paramedical professional under his/her responsibility, collection of non-objection and verification of inclusion and non-inclusion criteria, collection of patient characteristics and clinical data.

b) On the same day as inclusion, performance of step 1 "Screening": dispensing of the 10-item EAT-10 screening questionnaire by a paramedical professional

3) If EAT-10 score < 2: End of patient participation.
4) Completion of step 2 if EAT-10 score ≥ 2: Implementation (within three days of screening) by the health care team of upper airway protection measures appropriate to each patient.

Completion of the following checklist:

- Postural maneuvers (sitting eating, chin down, +/- head turned towards the paralysed limb, +/- double swallow, +/- Mendelsohn maneuver, +/- forced swallow, +/- (super)supraglottic swallow),

- Hygienic and dietary rules (eliminate risky foods, adapt fluids, take time, drink between sips, avoid distraction),

- Food textures (liquid, very slightly thick, slightly thick, moderately smooth/mixed smooth, mixed/pureed, ground, swallowing specific soft, normal).

5) Collection of the satisfaction and difficulties encountered by the involved allied health professionals with the program (online supplemental table 1).

Strategies for achieving adequate participant enrolment will regularly be implemented using formal (newsletters, posters, meetings) and informal methods to reach target sample size/

review only

233 **Table 1: DYSPHAGING-pilot study: flow diagram**

Visits	V1	V2	End of the implementation of the measures
**Time of evaluation**	**Inclusion**	**End of inclusion**	**End of the study**
**PATIENT**			
Information notice	X		
Collection of non opposition	X		
Inclusion and exclusion criteria	X		
Population demographics[1]	X		
Nutritional risk factors[2]	X		
Functionnal independendence (ADL, IADL)	X		
Sarcopenic dysphagia risk factors[3]	X		
Sarcopenic dysphagia screening (EAT-10)	X		
Airway protection measures[4]		X	
**CARE TEAM**			
Characteristics of the health care staff			X
Satisfaction questionnaire : Likert Scale			X

234
235 [1] Population demographics are age, gender, comorbidities (ICSR-G) and co-medications
236 [2] Nutritional risk factors are assessed by the Mini Nutritional Assessment® (MNA)
237 [3] Risk factors for sarcopenic dysphagia include undernutrition, neurocognitive impairment, overt lung infections
238 and chronic obstructive pulmonary disease (COPD)
239 [4] Upper airway protection recommendations are validated by the following 3 methods: postural maneuvers,
240 hygienic-dietary rules, textures within 3 days
241

242 ***Sample size calculation***

243 The program will be considered feasible, at the patient level, if the proportion of patients for
244 whom steps 1 and 2 are achievable is statistically higher than 35%, with an anticipated
245 proportion of 50% (= alternative hypothesis). Under theses hypotheses, and assuming 10% of
246 patients that might be non-evaluable, the inclusion of 102 patients will be necessary to achieve
247 90% power to show that the program is feasible (one-sided alpha risk of 5%). The included
248 patients will be analyzed according to the intention-to-treat principle.

For peer review only

Data management and statistical analyses

A CRA ensures proper study execution, data collection, and reporting. Inconsistencies will be reported to the study investigators in order to decide whether the data should be corrected or considered as missing. Adverse health events will be reported to regulatory authorities according to the legislation in force, provided they are aligned with the study's judgment criteria (inhalation/aspiration pneumonia, weight loss, death from any cause). Any changes in the data will be reported. A detailed statistical analysis plan will be drafted before the database is frozen. It will consider any changes in the protocol or unexpected events during the study that have an impact on the analyses presented above. Planned analyses may be completed in line with the study objectives. The analyses will be carried out by an independent statistician with the latest version of the SAS version 9.4 (SAS Institute, Cary, North Carolina) and R (R Core Team. R Foundation for Statistical Computing, Vienna, Austria. URL https:// www.R-project.org/) softwares environment. No intermediate analysis is scheduled.

Descriptive analyses

A flow diagram will describe the data available for the patient population at baseline and during each follow-up visit. Eligibility criteria for treated patients will be verified, as well as follow-up and end of study visits. Reasons for premature end of study will be provided. Characteristics of the study population, numbers and proportions of missing values will be reported. Patient characteristics will be described using mean and SD or median and IQR for quantitative variables, and frequencies and distribution for categorical variables. A comparison of baseline characteristics between patients with complete follow-up and those with attrition will be performed. Analyses will be performed on the available data, without imputation for missing data.

For peer review only - http://bmjopen.bmj.com/site/about/guidelines.xhtml

Primary analysis

The proportion of patients for whom steps 1 and 2 of the DYSPHAGING form in performed in the 3 days of inclusion will be assessed along with its corresponding 95% confidence interval. Patients for whom information on the completion of steps 1 and 2 is not available will be considered as not having completed these steps.

Secondary analyses

Analyses of the questionnaire for allied health professionals

Analyses will be performed independently using descriptive analyses for quantitative data using mean and SD or median and IQR for Likert scales; overt questions will be reported according to a flat analysis. The analysis of factors associated with sarcopenic dysphagia will be performed by logistic regression. Univariate analyses will be followed by multivariable analyses.

Confidentiality

Correspondence tables will be kept in a separate file that does not contain clinical data. The access to the nominative information is protected by a password, and confidentiality is guaranteed by the study.

Protocol amendments

A substantial protocol amendment was accepted by the ethics committee on December 13, 2023, to allow the inclusion of patients under guardianship, provided the oral or written consent of their legal guardian. Any important additional modification requiring a new ethics committee

For peer review only

approval will be communicated in future publications. Any potential impact of protocol modifications on the results will be discussed as appropriate.

Trial status

Patient enrolment began in May 2023. Data are currently being collected.

Patient and public involvement

The information letter and consent form for the study were reviewed by a patient partner.

Discussion

Discussion of the intervention

Despite growing interest in screening for swallowing disorders, ~~there is no~~ standardized method on which consensus has been reached (1) are not actually implemented in usual care (5). The main limitations include the heterogeneity of its presentations, the large number of etiologies, the poor reproducibility or complexity of screening processes and the need for a clinical confirmation by either a speech specialist or an ear, nose and throat physician. The absence of standardized procedure may lead to disjoined communications between hospital staffs and family carers, leading to suboptimal care, crispation and frustration (5). In addition, the need for a clinical confirmation of the swallowing problem may postpone the application of prevention procedures.

The aim of the DYSPHAGING approach is to bring together all the care providers around the patient, to ensure a multi-disciplinary approach, to use all the time spent with the patient to extract as much relevant information as possible, and to apply as soon as possible, before any clinical confirmation, basic safety measures with the help of a simple and schematic iconography. We believe that the screening and preventive measures proposed by this protocol

For peer review only

are appropriate for the healthcare providers working in various geriatric sectors, despite the heterogeneity of the situations encountered in this population. Moreover, the simplicity of the form helps to standardize practices, particularly in a context of high team turnover and may limit the risk of erosion in the application of protection measures, which nevertheless persists. In the future, the DYSPHAGING form is expected to be more widely diffused to caregivers and more generally all care providers, to reach ambulatory care. Due to its simple design, the tool is expected to allow a sharing of upper airway protection measures with the continuum of care providers around the patient, favoring adherence over time (13).

Discussion of the trial design

The main aim of this study is to assess the feasibility of screening and various preventive measures. The cutoff value of EAT10 of 2 was chosen to favor sensitivity over specificity, even if a recent meta-analysis argued for a better diagnostic accuracy with a cutoff value of 3 (14), as the DYSPHAGING form was focused more on screening than diagnosis (12). It is therefore essential to gather information on the non-implementation of the first steps, to understand the obstacles to the adoption of these initiatives. To simplify the research process and favor adherence by the teams, the primary outcome of the study was intentionally defined as the simplest possible, as the completion of steps 1 and 2 of the protocol, ie the follow-up ends after 3 days of patients' inclusion. Consequently, the statistical hypothesis did not include any a priori estimation of the rate of patients with an EAT10 score≥2 in the studied population, and this information will be of importance in the design of future trials. However, the trial design does not provide any longer term follow up of either the maintenance of the protective measures over time or the consequences of oral dysphagia (malnutrition, medical complications, etc), that would have been of interest for exploratory purposes. As healthcare staff are at the center of diagnosis and care, it is essential to understand the barriers and obstacles they face, by assessing

For peer review only

much feedback as possible. Particular attention was paid to the satisfaction of care providers in giving feedback about their training and the work tool. Emphasis was placed on assessing their satisfaction and the ergonomics of the tools made available to them, using a dedicated questionnaire. Future steps in the DYSPHAGING program of research will have to focus both on the implementation of the DYSPHAGING form in ambulatory care and on satisfaction of the other stakeholders with its ergonomics (patient, caregivers, care providers at home).

The galenic formulation and drug class will also be analyzed with care, as iatrogenicity is omnipresent in the geriatric population.

We hope to highlight the various difficulties encountered during this pilot study in order to draw the necessary conclusions for a larger-scale study.

Ethics and dissemination

The study sponsor is the Hospices Civils de Lyon, responsible for study insurance and pharmacovigilance. The study protocol (V1) was approved by the ethics committee on February 15, 2023; an amended version (V2) was approved on December 13, 2023 and covers all sites involved in this study. The research will be carried out in accordance with the Helsinki Declaration and International Conference on Harmonisation-Good Clinical Practice Guidelines. The trial protocol fulfils the SPIRIT 2013 checklist (online supplementary table 1) and WHO trial registration data set (online supplementary table 2). The study complies with the principles of the data protection act in France and with the GDPR in force in Europe. Each investigator must collect an oral informed consent at the beginning of the procedure. This consent is retained in the patient's medical chart. The patient can stop participation in the study at any time with an oral instruction given to the investigator or CRA. Patients will be informed of additional amendments according to the law in force. The results of the primary and secondary objectives will be published in peer-reviewed journals. All authors of future publications will have to meet

For peer review only

the criteria for authorship stated in the Uniform Requirements for Manuscripts Submitted to Biomedical Journals by the International Committee of Medical Journal Editors.

Declaration of interests

The authors declare that they have no conflicts of interest.

Access to data and Dissemination policy

The final data set of the DYSPHAGING pilot study will be available upon reasonable request after the publication of the primary objective. Data requests can be submitted to the corresponding author.

Ancillary and post-trial care

None.

Acknowledgements: The authors acknowledge the teams of Lyon Sud Hospital, Pierre Garraud Hospital who contribute to patient enrolment in this study. The authors would like to thank the Centre de Recherche Clinique (Clinical Research Center) Vieillissement Cerveau Fragilité and the Direction à la Recherche en Santé (Health Research Department) of the Hospices Civils de Lyon for their valuable help in trial design and conduct.

Contributors: OD, STdM, NMD, FS, ZNS, CH, LG, KG, SM, MC, MM and CF participated to the trial design conception. KG, STdM, AS, DD and CF managed fundraising and grant follow-up. OD led the drafting of the manuscript. All authors critically reviewed and approved the final version of the protocol.

Funding: This work was supported by the Institut Nutrition (Prix de l'Institut Nutrition 2021) and the Fondation de l'Avenir (Grant N°MLHR2023-89).

For peer review only

398 *Ethics approval:* The study protocol (V1) was approved by the Ile de France VII ethics committee on February 15, 2023 (N° 23.00016.0000172_AF_15022023); an amended version (V2) was approved on December 13, 2023 (N° 23.00016.0000172-MS01_AF_20231213) and covers all sites involved in this study.

Word count: 3,617

For peer review only

References

1. Baijens LW, Clavé P, Cras P, Ekberg O, Forster A, Kolb G, et al. European Society for Swallowing Disorders – European Union Geriatric Medicine Society white paper: oropharyngeal dysphagia as a geriatric syndrome. Clin Interv Aging. oct 2016;Volume 11:1403‑28.
2. Yardimci B, Aksoy SM, Ozkaya I, Demir T, Tezcan G, Kaptanoglu AY. Anthropometric measurements may be informative for nursing home-acquired pneumonia. Pak J Med Sci [Internet]. 7 mai 2016 [cité 22 oct 2023];32(3). Disponible sur: http://pjms.com.pk/index.php/pjms/article/view/9635
3. Okazaki T, Ebihara S, Mori T, Izumi S, Ebihara T. Association between sarcopenia and pneumonia in older people. Geriatr Gerontol Int. janv 2020;20(1):7‑13.
4. Serra-Prat M, Palomera M, Gomez C, Sar-Shalom D, Saiz A, Montoya JG, et al. Oropharyngeal dysphagia as a risk factor for malnutrition and lower respiratory tract infection in independently living older persons: a population-based prospective study. Age Ageing. 1 mai 2012;41(3):376‑81.
5. Anantapong K, Barrado-Martín Y, Nair P, Rait G, Smith CH, Moore KJ, et al. How do people living with dementia perceive eating and drinking difficulties? A qualitative study. Age Ageing. 11 sept 2021;50(5):1820‑8.
6. Belafsky PC, Mouadeb DA, Rees CJ, Pryor JC, Postma GN, Allen J, et al. Validity and Reliability of the Eating Assessment Tool (EAT-10). Ann Otol Rhinol Laryngol. déc 2008;117(12):919‑24.
7. Lechien JR, Cavelier G, Thill MP, Huet K, Harmegnies B, Bousard L, et al. Validity and reliability of the French version of Eating Assessment Tool (EAT-10). Eur Arch Otorhinolaryngol. juin 2019;276(6):1727‑36.
8. Rofes L, Arreola V, Mukherjee R, Clavé P. Sensitivity and specificity of the Eating Assessment Tool and the Volume-Viscosity Swallow Test for clinical evaluation of oropharyngeal dysphagia. Neurogastroenterol Motil. sept 2014;26(9):1256‑65.
9. Cichero JAY, Lam P, Steele CM, Hanson B, Chen J, Dantas RO, et al. Development of International Terminology and Definitions for Texture-Modified Foods and Thickened Fluids Used in Dysphagia Management: The IDDSI Framework. Dysphagia. avr 2017;32(2):293‑314.
10. Katz S. Studies of Illness in the Aged: The Index of ADL: A Standardized Measure of Biological and Psychosocial Function. JAMA. 21 sept 1963;185(12):914.
11. Lawton MP, Brody EM. Assessment of older people: self-maintaining and instrumental activities of daily living. The Gerontologist. 1969;9(3):179‑86.
12. Diagnostic de la dénutrition chez la personne de 70 ans et plus.
13. Osterberg L, Blaschke T. Adherence to Medication. N Engl J Med. 4 août 2005;353(5):487‑97.
14. Zhang P ping, Yuan Y, Lu D zhi, Li T ting, Zhang H, Wang H ying, et al. Diagnostic Accuracy of the Eating Assessment Tool-10 (EAT-10) in Screening Dysphagia: A Systematic Review and Meta-Analysis. Dysphagia. févr 2023;38(1):145‑58.

For peer review only

Figure Legends:

Figure 1: The DYSPHAGING Form (A: recto; B: verso)

Figure 2: Design of the DYSPHAGING-pilot study

Table 1: DYSPHAGING-pilot study: flow diagram

For peer review only

A

LAST NAME First name
Date of birth or label

DYSPHAGING

First Name – NAME of investigateur / healthcare professional
………..
Profession : ☐ Doctor ☐ Nurse ☐ Nursing assistant
Information note provided ☐ Yes ☐ No
Oral consent obtained ☐ Yes ☐ No Date : ……./……/………

1 SCREEN

To what extent are the following scenarios problematic for you ?

0_____1_____2_____3_____4
0 : No problem **4: severe problem**

1 – My swallowing problem has caused me to lose weight	
2 – My swallowing problem interferes with my ability to go out for meals.	
3 – Swallowing liquids takes extra effort.	
4 – Swallowing solids takes extra effort.	
5 – Swallowing pills takes extra effort.	
6 - Swallowing is painful.	
7 – The pleasure of eating is affected by my swallowing.	
8 – When i swallow food sticks in my throat.	
9 – I cough when i eat.	
10 – Swallowing is stressful.	

If score ≥ 2 : Implement protective maneuvers

B

PROTECT 2

1 – POSTURAL MANEUVERS

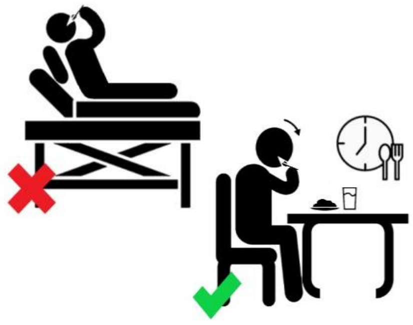

- ☐1 Sit while eating
- ☐2 Chin down
- ☐3 +/- head turned towards the paralysed limb
- ☐4 +/- double swallow
- ☐5 +/- Mendelsohn maneuver
- ☐6 +/- forced swallow
- ☐7 +/- (super) supraglottic swallow

2 – HYGIENIC AND DIETETIC RULES

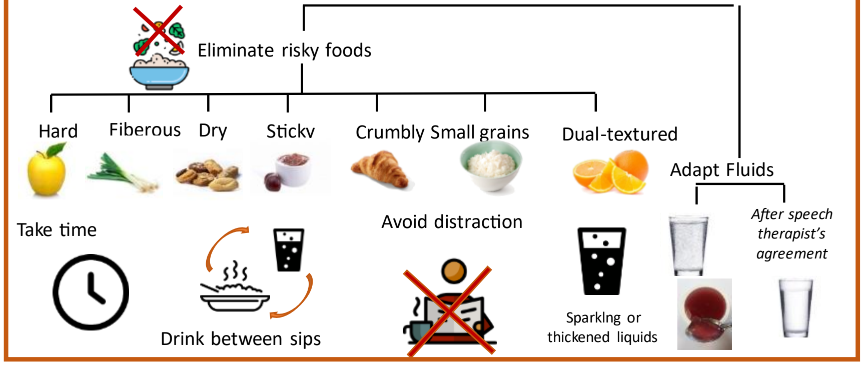

3 – FOOD TEXTURES

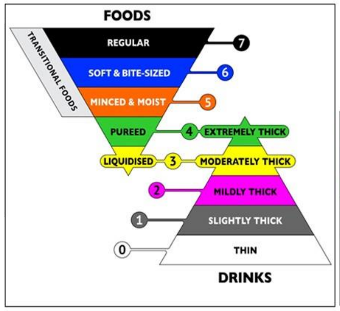

- ☐0 Thin
- ☐1 Slightly thick
- ☐2 Mildly thick
- ☐3 Moderately thick / Liquidised
- ☐4 Extremely thick / pureed
- ☐5 Minced & moist
- ☐6 Soft & bite-sized
- ☐7 Regular

International Dysphagia Diet Standardization Initiative [www.iddsi.org]).

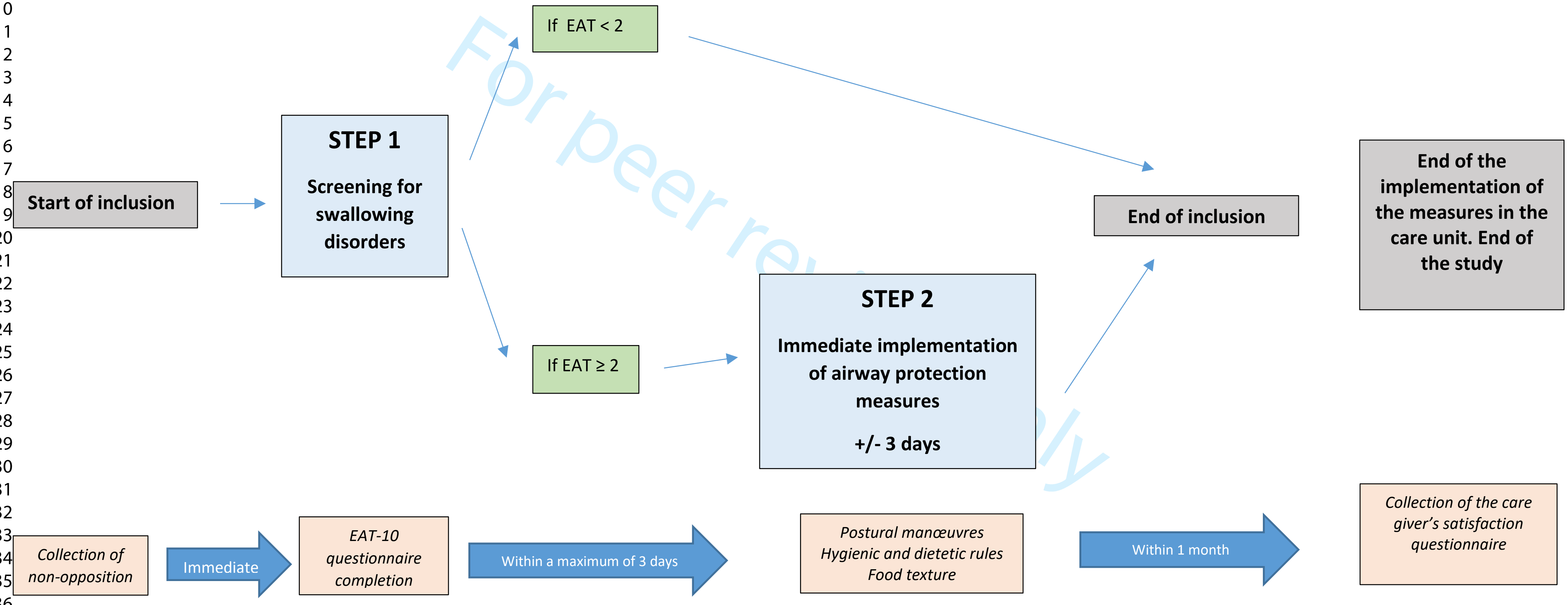
If EAT < 2
STEP 1
Screening for swallowing disorders
Start of inclusion
End of inclusion
End of the implementation of the measures in the care unit. End of the study
STEP 2
Immediate implementation of airway protection measures
+/- 3 days
If EAT ≥ 2
Collection of non-opposition
Immediate
EAT-10 questionnaire completion
Within a maximum of 3 days
Postural manœuvres
Hygienic and dietetic rules
Food texture
Within 1 month
Collection of the care giver's satisfaction questionnaire

Patient code : /__/ /__/ /__//__/ /__/ /__/ /__//__/
First letter : Last name then first name centre N° patient identification N°

Online supplementary document 1

ALLIED HEALTH PROFESSIONAL SECTION

Page 1 : Characteristics of the respondant

You are:

☐ Nurse

☐ Nursing Assistant

☐ Doctor

☐ Else : ……………………………………………………………………

Page 2 : Satisfaction questionnaire

If you take the presentation of the study as a whole

1- *Strongly disagree*
2- *Somewhat disagree*
3- *No opinion*
4- *Somewhat agree*
5- *Strongly agree*

	1	*2*	*3*	*4*	*5*
**Do you think the explanations are appropriate?**					
**Was the time allocated sufficient?**					
**Is the summary sheet clear?**					
**Do you think the illustrations are clear?**					

How would you rate the presentation session?
(useless = 0 ; very useful = 10) : ……………..

Did you find the procedure (DYSPHAGING form) simple and feasible to carry out in your current practice?

	1	*2*	*3*	*4*	*5*
**EAT-10 questionnaire ?**					
**Airway protection manœuvres ?**					
**Hygienic and dietary measures?**					
**Procedures for adapting textures?**					

How would you rate the DYSPHAGING form?
(useless = 0 ; very useful = 10) : ………..

For peer review only

Patient code : /__/ /__/ /__//__/ /__/ /__/ /__//__/
First letter : Last name then first name centre N° patient identification N°

Have you encountered any difficulties?

1 Not at all
2 Some
3 A lot

	1	2	3
**when presentating to the patient the information leaflet?**			
**For informing the patient's entourage?**			
**For collecting oral consent?**			
**For carrying out the EAT-10 questionnaire?**			
**For carrying out protection manoeuvres?**			

Have you encountered any difficulties (questions concerning paramedical research)

1 Not at all
2 Some
3 A lot

	1	2	3
**when presentating to the patient the information leaflet?**			
**For informing the patient's entourage?**			
**For collecting oral consent?**			
**For carrying out the EAT-10 questionnaire?**			
**For carrying out protection manoeuvres?**			

Would you have liked more information? No ☐ Yes ☐
If so, which ones? ……………………………………………………………………………………………
………
………
………

Which suggestions would you make to make the protocol more relevant to your practice?
……………………………………………………………………………………………
……
……………………………………………………………………………………………

For peer review only

Supplementary table 1: SPIR

SPIRIT

STANDARD PROTOCOL ITEMS: RECOMMENDATIONS FOR INTERVENTIONAL TRIALS

SPIRIT 2013 Checklist: Recommended items to address in a clinical trial protocol and related documents*

Section/item	ItemNo	Description	Reported on page #
**Administrative information**			
Title	1	Descriptive title identifying the study design, population, interventions, and, if applicable, trial acronym	1
Trial registration	2a	Trial identifier and registry name. If not yet registered, name of intended registry	1
	2b	All items from the World Health Organization Trial Registration Data Set	Online supplementary table 2
Protocol version	3	Date and version identifier	15 (Ethics and dissemination)
Funding	4	Sources and types of financial, material, and other support	16 (Funding)
Roles and responsibilities	5a	Names, affiliations, and roles of protocol contributors	1 (Authors' list)<br>16 (Contributors)
	5b	Name and contact information for the trial sponsor	15 (ethics and dissemination)

For peer review only

	5c	Role of study sponsor and funders, if any, in study design; collection, management, analysis, and interpretation of data; writing of the report; and the decision to submit the report for publication, including whether they will have ultimate authority over any of these activities	15 (ethics and dissemination)
	5d	Composition, roles, and responsibilities of the coordinating centre, steering committee, endpoint adjudication committee, data management team, and other individuals or groups overseeing the trial, if applicable (see Item 21a for data monitoring committee)	10 (Data management and statistical analyses) 15 (Ethics and dissemination)
**Introduction**			
Background and rationale	6a	Description of research question and justification for undertaking the trial, including summary of relevant studies (published and unpublished) examining benefits and harms for each intervention	4
	6b	Explanation for choice of comparators	N/A
Objectives	7	Specific objectives or hypotheses	5
Trial design	8	Description of trial design including type of trial (eg, parallel group, crossover, factorial, single group), allocation ratio, and framework (eg, superiority, equivalence, noninferiority, exploratory)	5
**Methods: Participants, interventions, and outcomes**			
Study setting	9	Description of study settings (eg, community clinic, academic hospital) and list of countries where data will be collected. Reference to where list of study sites can be obtained	6

For peer review only

Eligibility criteria	10	Inclusion and exclusion criteria for participants. If applicable, eligibility criteria for study centres and individuals who will perform the interventions (eg, surgeons, psychotherapists)	5
Interventions	11a	Interventions for each group with sufficient detail to allow replication, including how and when they will be administered	6
	11b	Criteria for discontinuing or modifying allocated interventions for a given trial participant (eg, drug dose change in response to harms, participant request, or improving/worsening disease)	6
	11c	Strategies to improve adherence to intervention protocols, and any procedures for monitoring adherence (eg, drug tablet return, laboratory tests)	N/A
	11d	Relevant concomitant care and interventions that are permitted or prohibited during the trial	N/A
Outcomes	12	Primary, secondary, and other outcomes, including the specific measurement variable (eg, systolic blood pressure), analysis metric (eg, change from baseline, final value, time to event), method of aggregation (eg, median, proportion), and time point for each outcome. Explanation of the clinical relevance of chosen efficacy and harm outcomes is strongly recommended	8 (Outcomes and measurements)
Participant timeline	13	Time schedule of enrolment, interventions (including any run-ins and washouts), assessments, and visits for participants. A schematic diagram is highly recommended (see Figure)	18 (Figure 2)
Sample size	14	Estimated number of participants needed to achieve study objectives and how it was determined, including clinical and statistical assumptions supporting any sample size calculations	10 (Sample size calculation)

Recruitment	15	Strategies for achieving adequate participant enrolment to reach target sample size	N/A
**Methods: Assignment of interventions (for controlled trials)**			**N/A**
Allocation:			
Sequence generation	16a	Method of generating the allocation sequence (eg, computer-generated random numbers), and list of any factors for stratification. To reduce predictability of a random sequence, details of any planned restriction (eg, blocking) should be provided in a separate document that is unavailable to those who enrol participants or assign interventions	-
Allocation concealment mechanism	16b	Mechanism of implementing the allocation sequence (eg, central telephone; sequentially numbered, opaque, sealed envelopes), describing any steps to conceal the sequence until interventions are assigned	-
Implementation	16c	Who will generate the allocation sequence, who will enrol participants, and who will assign participants to interventions	-
Blinding (masking)	17a	Who will be blinded after assignment to interventions (eg, trial participants, care providers, outcome assessors, data analysts), and how	-
	17b	If blinded, circumstances under which unblinding is permissible, and procedure for revealing a participant's allocated intervention during the trial	-
**Methods: Data collection, management, and analysis**			

Data collection methods	18a	Plans for assessment and collection of outcome, baseline, and other trial data, including any related processes to promote data quality (eg, duplicate measurements, training of assessors) and a description of study instruments (eg, questionnaires, laboratory tests) along with their reliability and validity, if known. Reference to where data collection forms can be found, if not in the protocol	9, 1à, Table 1
	18b	Plans to promote participant retention and complete follow-up, including list of any outcome data to be collected for participants who discontinue or deviate from intervention protocols	N/A
Data management	19	Plans for data entry, coding, security, and storage, including any related processes to promote data quality (eg, double data entry; range checks for data values). Reference to where details of data management procedures can be found, if not in the protocol	10 (Data management and statistical analyses)
Statistical methods	20a	Statistical methods for analysing primary and secondary outcomes. Reference to where other details of the statistical analysis plan can be found, if not in the protocol	10 (Data management and statistical analyses)
	20b	Methods for any additional analyses (eg, subgroup and adjusted analyses)	N/A
	20c	Definition of analysis population relating to protocol non-adherence (eg, as randomised analysis), and any statistical methods to handle missing data (eg, multiple imputation)	11 (Descriptive analyses)
**Methods: Monitoring**			

Data monitoring	21a	Composition of data monitoring committee (DMC); summary of its role and reporting structure; statement of whether it is independent from the sponsor and competing interests; and reference to where further details about its charter can be found, if not in the protocol. Alternatively, an explanation of why a DMC is not needed	N/A
	21b	Description of any interim analyses and stopping guidelines, including who will have access to these interim results and make the final decision to terminate the trial	N/A
Harms	22	Plans for collecting, assessing, reporting, and managing solicited and spontaneously reported adverse events and other unintended effects of trial interventions or trial conduct	10 (Data management and statistical analyses)
Auditing	23	Frequency and procedures for auditing trial conduct, if any, and whether the process will be independent from investigators and the sponsor	N/A
**Ethics and dissemination**			
Research ethics approval	24	Plans for seeking research ethics committee/institutional review board (REC/IRB) approval	15 (Ethics and dissemination)
Protocol amendments	25	Plans for communicating important protocol modifications (eg, changes to eligibility criteria, outcomes, analyses) to relevant parties (eg, investigators, REC/IRBs, trial participants, trial registries, journals, regulators)	12 (Protocol amendments)
Consent or assent	26a	Who will obtain informed consent or assent from potential trial participants or authorised surrogates, and how (see Item 32)	15 (Ethics and dissemination)
	26b	Additional consent provisions for collection and use of participant data and biological specimens in ancillary studies, if applicable	N/A

For peer review only - http://bmjopen.bmj.com/site/about/guidelines.xhtml

Confidentiality	27	How personal information about potential and enrolled participants will be collected, shared, and maintained in order to protect confidentiality before, during, and after the trial	12 (Confidentiality)
Declaration of interests	28	Financial and other competing interests for principal investigators for the overall trial and each study site	15 (Declaration of interests)
Access to data	29	Statement of who will have access to the final trial dataset, and disclosure of contractual agreements that limit such access for investigators	16 (Access to data and dissemination policy)
Ancillary and post-trial care	30	Provisions, if any, for ancillary and post-trial care, and for compensation to those who suffer harm from trial participation	N/A
Dissemination policy	31a	Plans for investigators and sponsor to communicate trial results to participants, healthcare professionals, the public, and other relevant groups (eg, via publication, reporting in results databases, or other data sharing arrangements), including any publication restrictions	16 (Access to data and dissemination policy)
	31b	Authorship eligibility guidelines and any intended use of professional writers	15 (Dissemination policy), N/A
	31c	Plans, if any, for granting public access to the full protocol, participant-level dataset, and statistical code	16 (Acces to data and dissemination policy)
**Appendices**			
Informed consent materials	32	Model consent form and other related documentation given to participants and authorised surrogates	Fig. 1 DYSPHAGING Form
Biological specimens	33	Plans for collection, laboratory evaluation, and storage of biological specimens for genetic or molecular analysis in the current trial and for future use in ancillary studies, if applicable	N/A

For peer review only

*It is strongly recommended that this checklist be read in conjunction with the SPIRIT 2013 Explanation & Elaboration for important clarification on the items. Amendments to the protocol should be tracked and dated. The SPIRIT checklist is copyrighted by the SPIRIT Group under the Creative Commons "Attribution-NonCommercial-NoDerivs 3.0 Unported" license.

For peer review only

Supplementary Table 2: World Health Organization Trial Registration Data Set.

**Data category**	**Information**[32]
Primary registry and trial identifying number	ClinicalTrials.gov<br>NCT05734586
Date of registration in primary registry	8 February, 2023
Secondary identifying numbers	69HCL22_0474
Source(s) of monetary or material support	Hospices Civils de Lyon, France
Primary sponsor	Hospices Civils de Lyon, France
Secondary sponsor(s)	N/A
Contact for public queries	Marion MERDINIAN, MD<br>Tel: 00 33 4 78 86 56 83<br>E-mail: marion.merdinian@chu-lyon.fr
Contact for scientific queries	Claire FALANDRY, MD, PhD<br>Tel: 00 33 4 78 86 66 34<br>E-mail: claire.falandry@chu-lyon.fr
Public title	Screening for Sarcopenic Dysphagia and the Implementation of Measures to Prevent Its Complications in Geriatric Patients [DYSPHAGING-PILOT]
Scientific title	Feasibility Study of Screening for Sarcopenic Dysphagia and the Implementation of Measures to Prevent Its Complications in Geriatric or Institutionalized Patients Aged ≥ 70 Years.
Countries of recruitment	France

For peer review only

**Data category**	**Information**[32]
Health condition(s) or problem(s) studied	Swallowing Disorder, Sarcopenic Dysphagia
Intervention(s)	Other: EAT-10 (Eating assessment Tool) screening questionnaire<br>After inclusion, issuance of the EAT-10 screening questionnaire for swallowing disorders by the healthcare team<br>Procedure: Protective measures for the upper airways<br>In the event of an EAT ≥2 score, immediate implementation or within three days by the healthcare team of protective measures for the upper airways in 3 sectors:<br>1: Postural maneuvers; 2: Hygienodietetic rules; 3: Food textures
Key inclusion and exclusion criteria	Inclusion Criteria:<br>• Patient aged ≥ 70 years,<br>• Patient affiliated to a social security system,<br>• Patient hospitalized in the health sector or in a medico-social institute,<br>• Patient informed of the study (information leaflet provided) and having orally signified their consent to inclusion in the study.<br>Exclusion Criteria:<br>• Patient unable to feed orally,<br>• Patient with an active pathology responsible for acute swallowing disorders (< 3 months) (neurodegenerative pathology with predominant motor impairment such as Charcot's disease, stroke, ENT disease).<br>• Patient unable to answer the questionnaire.
Study type	Interventional<br>Allocation: N/A<br>Intervention model: parallel assignment<br>Masking: None (Open Label)<br>Primary purpose: Other<br>Phase II
Date of first enrolment	June 1st,2023
Target sample size	102
Recruitment status	Recruiting
Primary outcome(s)	Proportion of complete achievement of steps 1 and 2 [Time Frame: Three days]<br>The judgment criterion is validated if<br>1. Stage 1 is performed and the EAT-10 < 2 or if<br>2. Stage 1 is performed with an EAT-10 ≥ 2 and stage 2 is performed within 3 days after stage 1.

For peer review only

Data category	Information[32]
Key secondary outcomes	• Percentage of eligible patients refusing to participate in the study [Time Frame: 18 months]<br>○ Number of eligible patients who refused to participate in the study<br>• Age, gender, comorbidities (CIRS-G), autonomy (ADL, IADL), co-medications [Time Frame: 19 months]<br>○ Patient characteristics will be collected at each site at the end of the study by a clinical research assistant based on their medical records.<br>• Rate of partial completion of the protocol [Time Frame: 19 months]<br>○ Proportion of non-performance of step 1 and/or step 2 within the time limit. Proportion of steps 2 carried out incompletely), description of the reasons<br>• Diagnosis of undernutrition and/or neurocognitive disorders and/or patent lung infection and/or COPD described in the patient's medical file, nutritional risk situation assessed by the Mini Nutritional Assessment® (MNA) [Time Frame: 19 months]<br>○ Patient characteristics will be collected at each site at the end of the study by a clinical research assistant based on their medical records.<br>• Composition and disciplines of the care team [Time Frame: 19 months]<br>○ At the end of the study, all data on the each allied health professionals will be collected on the dysphaging sheet<br>• Satisfaction of the care team (Likert scale). [Time Frame: 19 months]<br>○ At the end of the study, each allied health professionals who has been involved in the care of at least one patient will fill out a satisfaction questionnaire.

For peer review only
